# Author Correction: On the longevity and inherent hermeticity of silicon-ICs: evaluation of bare-die and PDMS-coated ICs after accelerated aging and implantation studies

**DOI:** 10.1038/s41467-025-57175-0

**Published:** 2025-02-24

**Authors:** Kambiz Nanbakhsh, Ahmad Shah Idil, Callum Lamont, Csaba Dücső, Ömer Can Akgun, Domonkos Horváth, Kinga Tóth, Domokos Meszéna, István Ulbert, Federico Mazza, Timothy G. Constandinou, Wouter Serdijn, Anne Vanhoestenberghe, Nick Donaldson, Vasiliki Giagka

**Affiliations:** 1 Department of Microelectronics, Faculty of Electrical Engineering, Mathematics and Computer Science, Delft University of Technology, Delft, The Netherlands; 2 Department of Medical Physics and Biomedical Engineering, University College London, London, UK; 3 Department of Electrical & Electronic Engineering, Imperial College London, London, UK; 4 UK Dementia Research Institute, Care Research and Technology Centre, London, UK; 5 Mint Neurotechnologies Ltd, London, UK; 6 Centre for Energy Research, HUN-REN, Budapest, Hungary; 7 Nikhef - Dutch National Institute for Subatomic Physics, Amsterdam, the Netherlands; 8 Research Centre for Natural Sciences, Institute of Cognitive Neuroscience and Psychology, HUN-REN, Budapest, Hungary; 9 Pazmany Peter Catholic University, Faculty of Information Technology and Bionics, Budapest, Hungary; 10 Department of Neuroscience, Erasmus Medical Center, Rotterdam, The Netherlands; 11 School of Biomedical Engineering & Imaging Sciences, King’s College London, London, UK; 12 Department of System Integration and Interconnection Technologies, Fraunhofer Institute for Reliability and Microintegration IZM, Berlin, Germany

**Keywords:** Implants, Polymers, Electronic devices, Biomedical engineering, Electrical and electronic engineering

Correction to: *Nature Communications* 10.1038/s41467-024-55298-4, published online 02 January 2025

In this article the following sentence was omitted from the acknowledgements section, ‘This research was funded by the following projects: Project CANDO (Controlling Network Dynamics with Optogenetics), funded by UK EPSRC (grant ref: NS/A000026/1) and the Wellcome Trust (contract ref: 102037/Z/13/Z)’. The original article has been corrected.

