# Supplementary material for: On the longevity and inherent hermeticity of silicon-ICs: evaluation of bare-die and PDMS-coated ICs after accelerated aging and implantation studies
Source: Nat Commun. 2025 Jan 2;16:12. doi: 10.1038/s41467-024-55298-4 (PMC11697292; doi:10.1038/s41467-024-55298-4)
Supplement: Supplementary file 1 — Supplementary Information [file 41467_2024_55298_MOESM1_ESM.pdf]

Supplementary Information

On the Longevity and Inherent Hermiticity of Silicon-ICs: Evaluation of Bare-Die and PDMS-Coated ICs After Accelerated Aging and Implantation Studies

*Kambiz Nanbakhsh[1], Ahmad Shah Idil[2,3,4,5], Callum Lamont[2], Csaba Dücső[6], Ömer Can Akgun[1,7], Domonkos Horváth[8,9], Kinga Tóth[8,9], Domokos Meszéna[8,9], István Ulbert[8,9], Federico Mazza[3], Timothy G. Constandinou[3,4,5], Wouter Serdijn[1,10], Anne Vanhoestenberghe[2,11], Nick Donaldson[2], Vasiliki Giagka[1,12] **

*Corresponding author: Dr. Vasiliki Giagka, v.giagka@tudelft.nl

[1]*Department of Microelectronics, Faculty of Electrical Engineering, Mathematics and Computer Science, Delft University of Technology, Delft, The Netherlands.*

[2]*Department of Medical Physics and Biomedical Engineering, University College London, London, United Kingdom.*

[3]*Department of Electrical & Electronic Engineering, Imperial College London, United Kingdom.*

[4]*UK Dementia Research Institute, Care Research and Technology Centre, London, United Kingdom.*

[5]*Mint Neurotechnologies Ltd, London, United Kingdom.*

[6]*Centre for Energy Research, HUN-REN, Budapest, Hungary*

[7]*Nikhef - Dutch National Institute for Subatomic Physics, Amsterdam, the Netherlands*

[8]*Research Centre for Natural Sciences, Institute of Cognitive Neuroscience and Psychology, HUN-REN, Budapest, Hungary.*

[9]*Pazmany Peter Catholic University, Faculty of Information Technology and Bionics, Budapest, Hungary*

[10]*Department of Neuroscience Erasmus Medical Center, Rotterdam, The Netherlands.*

[11]*School of Biomedical Engineering & Imaging Sciences King's College London.*

[12]*Department of System Integration and Interconnection Technologies, Fraunhofer Institute for Reliability and Microintragration IZM, Berlin, Germany.*

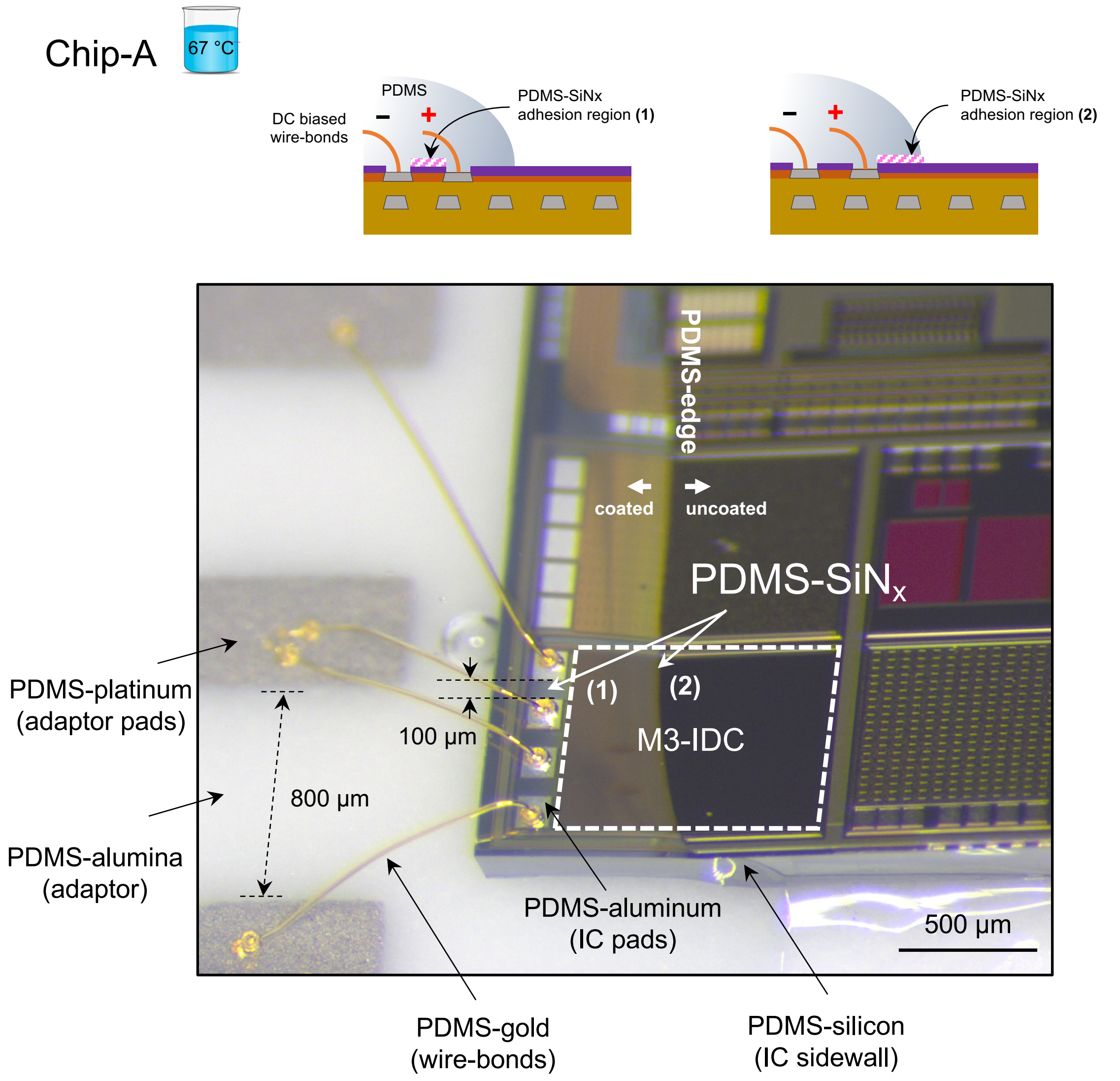

Figure S1. A tilted optical micrograph of a representative wire-bonded M3-IDC test structure (Chip-A) used for the accelerated *in vitro* aging study demonstrating the 6 critical PDMS interface bonds on the test structure. Schematics are not to scale.

Supplementary Note 1: PDMS-device interfaces. De-bonding of PDMS could affect the electrical measurements during the *in vitro* accelerated aging study. Figure S1 presents the 6 PDMS interface bonds on the test structures. The most critical interface is the PDMS-SiN_x interface bond and is composed of two regions: (1) the region between the IC wire-bond pads and (2) the region extending to the PDMS-edge. Both interface regions are crucial for maintaining a stable electrical performance. The interface at the PDMS-edge is directly exposed to phosphate buffered saline (PBS) solution where interfacial debonding between the PDMS and SiN_x will allow lateral ingress of ionic liquid. All other interfaces are protected from the ionic fluid by PDMS. Nevertheless, due to the moisture permeability of PDMS, these interfaces will be subjected to moisture (gaseous water). At the regions between the IC pads, debonding of the PDMS-SiN_x interface would allow the condensation of the diffused moisture, resulting in shunt water-leakage paths between the pads. Given the narrow ~100 µm gap between these pads, PDMS de-bonding in this region will impact the electrical performance. Note that the PDMS also acts as an electrical insulator, both between the wire bonds and between the wire bonds and the PBS solution. Therefore, its bulk electrical stability is essential for maintaining a stable long-term electrical performance.

Supplementary Note 2: The interdigitated capacitor (IDC) structures were created either on the top-most metal layer available in each process, i.e., Metal-4 (M4) or Metal-6 (M6), for Chip-A and Chip-B, respectively, or on the metal layer directly below, i.e., Metal-3 (M3) or Metal-5 (M5). For the second case, two variations were included: 1) leaving the top-most metal unused; or, 2) using the top metal to protect the underlying IDC, creating a shield (SH) on top, with the intention that it would act as a metal barrier, further delaying moisture or ion penetration. The shield is not a continuous layer of metal but is a slotted (3 μm x 30 μm and 5 μm x 5 μm slots for Chip-A and B, respectively) to comply with the metal density rules specified by the foundries. In both Chip-A and B, the IDCs were designed to have a relatively large area (700 μm x 600 μm). The gap between the comb metals in the IDCs is 0.6 μm. The larger IDC area would allow a larger area of the IC passivation layers to be evaluated for possible defects. Increasing the area would also increase the capacitance, making it large enough to be measurable with the Solartron Modulab at the lowest frequency (0.01 Hz). However, increasing the capacitance value of the IDC test structure would lower the sensitivity threshold of the impedance characterization. Here, the IDCs were designed to have a capacitance of ~ 60 - 70 pF. More detailed information on the IDC design can be found in [1].

Supplementary Note 3: Measurements to quantify the thickness of the PDMS coating on the **thickest region** for the *in vitro* and *in vivo* samples. Measurements were done using Leica DCM8 3D surface metrology microscope.

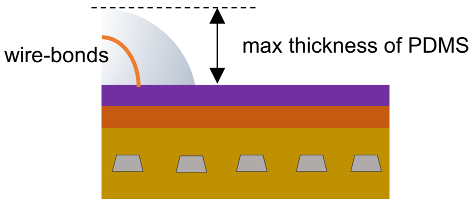

Sample	Thickness (μm)
In vitro sample #1 (with wire-bonds)	793
In vitro sample #2 (with wire-bonds)	631
In vitro sample #3 (with wire-bonds)	750
In vivo sample #1 (without wire-bonds)	284
In vivo sample #2 (without wire-bonds)	310

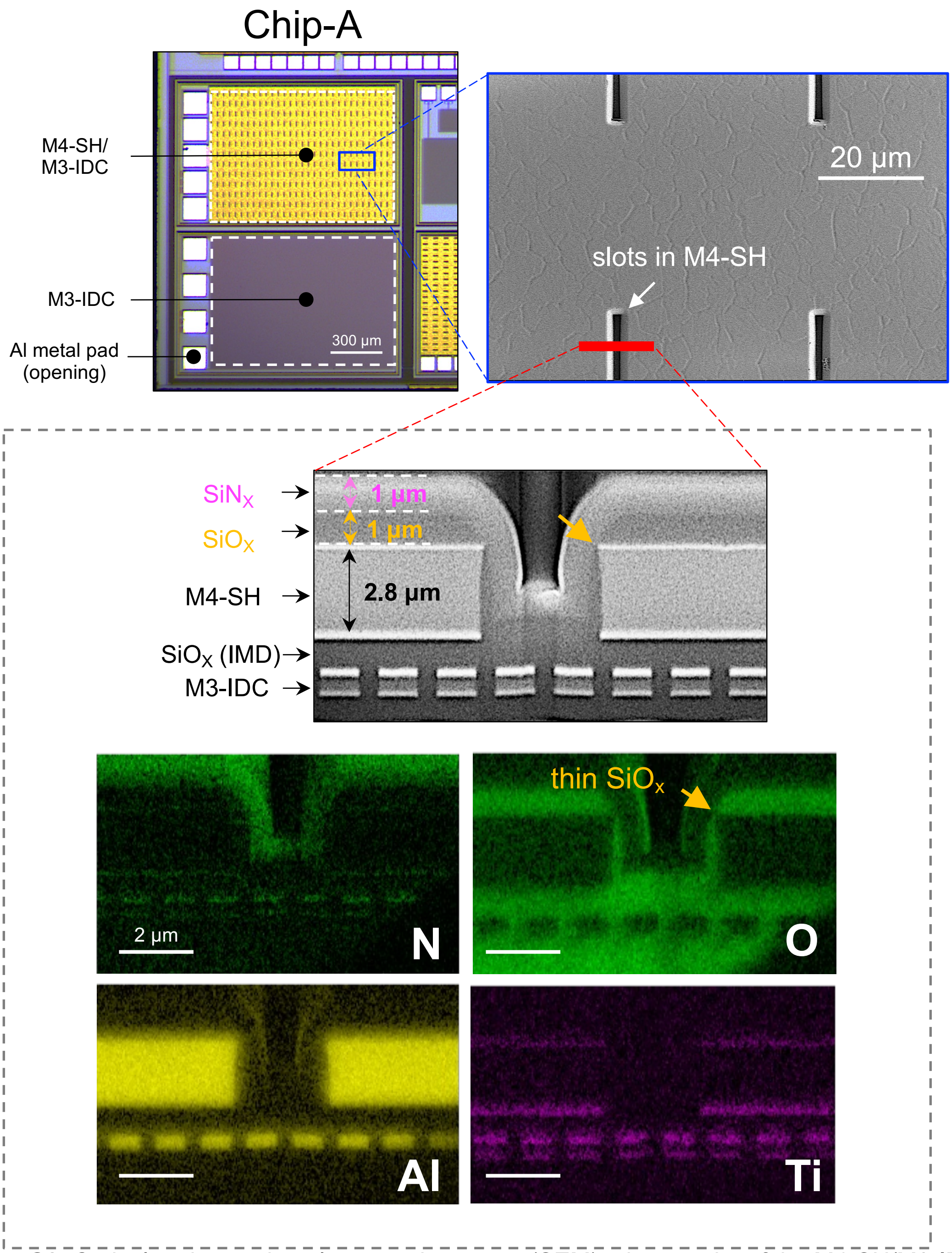

Figure S2. Optical and scanning electron microscopy (SEM) micrographs of the M4-SH/M3-IDC test structure (Chip-A), shield (SH) layer in Metal-4 and interdigitated capacitor (IDC) in Metal-3),. Top right SEM depicts the surface microtopography created due to the slots in metal 4 (top metal). Slots are created in the metal layer to obey metal density rules defined by the IC foundry. Red line indicates the focused ion beam (FIB) cut for creating the cross section. SEM micrograph and energy dispersive X-ray (EDX) elemental mapping of the cross section showing the top material stack of the IC: SiN_X/SiO_X passivation layers, Metal-4 shield layer and the M3-IDC structure (scale bar is similar for all elemental maps). The presence of the top metal creates poor conformality in the passivation layers, specially resulting in a much thinner SiO_X passivation layer (<100 nm) on the edges of Metal-4.

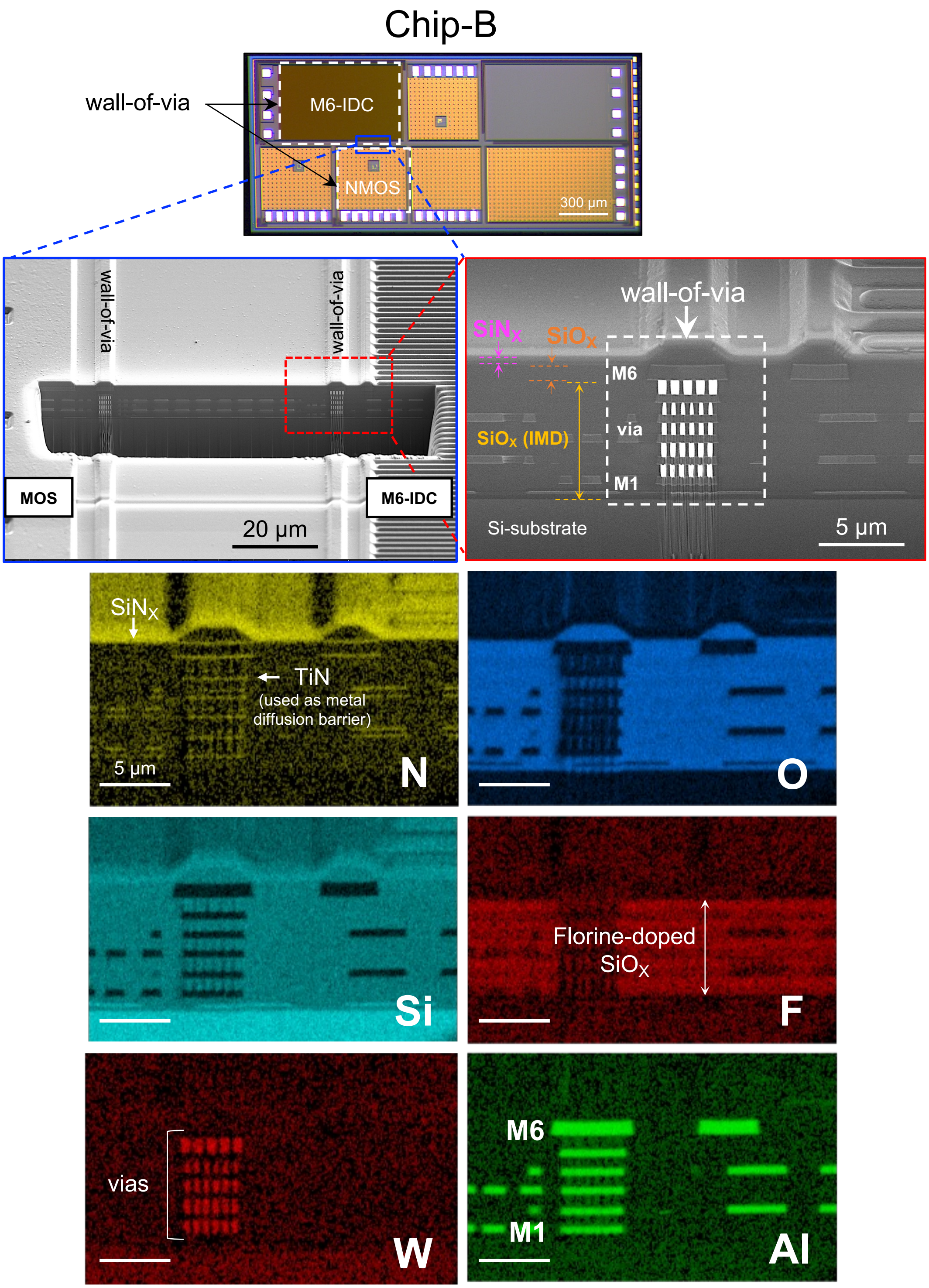

Figure S3. Optical and electron micrographs with EDX elemental mapping of wall-of-via on a Chip-B IC. White dashed lines in optical micrograph show the wall-of-via (WoV) structure implemented around each test structure. Surface and cross-sectional SEM images show the WoV around the negative metal oxide semiconductor (NMOS) transistor and M6-IDC structures. Surface topography is seen as a result of using top metallization (M6). EDX elemental mapping shows the material stack of Chip-B (scale bar is similar for all elemental maps). The WoV is implemented using M1 to M6 aluminum (Al) metallization and tungsten (W) vias. All aluminum metallization is sandwiched between a top and bottom thin titanium nitride (TiN) layer, which is used as a metal diffusion barrier [4]. The fluorine (F) intensity in the intermetallic dielectric (IMD) layers shows that the IMD layers for Chip-B are fluorine-doped silicon oxide (SiOF) to reduce the dielectric constant of the layers (*k* ~ 3.5).

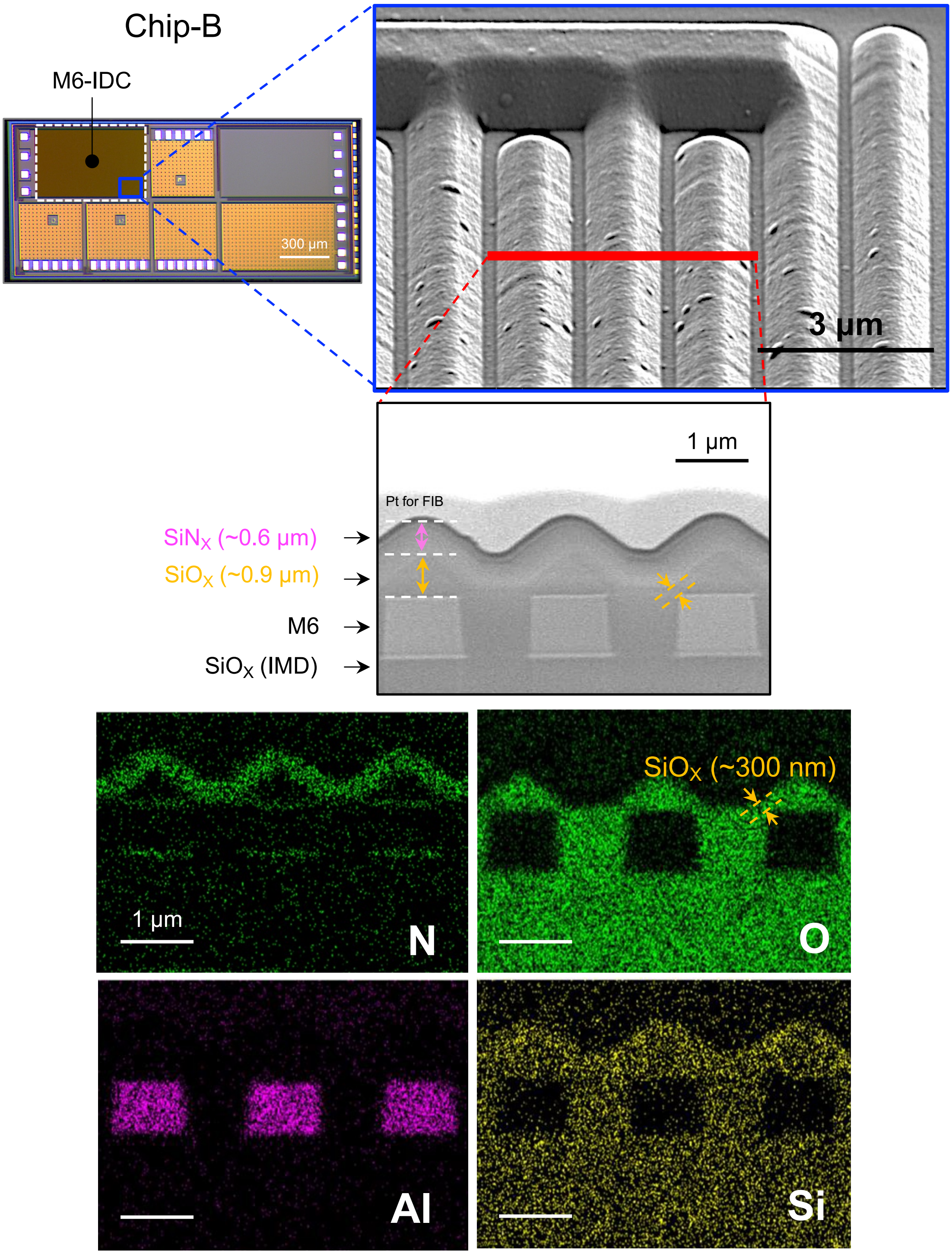

Figure S4. Optical and electron micrographs of a M6-IDC test structure (Chip-B). Tilted SEM image show surface microtopography created due to the use of the top metal layer (M6) for the interdigitated test structure. Red line indicates the FIB cut for cross sectioning. SEM and EDX elemental mapping of the cross section depicts the top few layers showing the SiN_X and SiO_X passivation. Due to the presence of the top metallization, poor conformality of the oxide passivation can be observed resulting in a thinner (~300 nm) SiO_X passivation on the edges of the metal fingers.

Table S1: Chip-A* test structures with specifications: interdigitated capacitor (IDC) test structures implemented using a 0.35 μm 4-metal process with thick top metal.

Structure	Image	Design parameters
M3-IDC<br>(1.15 x 0.85 mm)	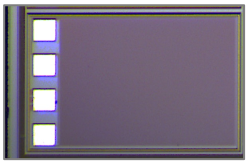	IDC implemented in Metal 3<br>Pads in Metal 4 (top metal)<br>Bond pads size: 150 x 150 μm^2<br>Passivated everywhere except pads<br>Wall of via (WoV) around the whole structure<br>Finger width = 1 μm<br>Finger gap = 0.6 μm<br>Number of fingers = 716 (358 each electrode)<br>Finger length = 844 μm
M4-SH/M3-IDC<br>(0.97 x 0.68 mm)	**Shielded**<br>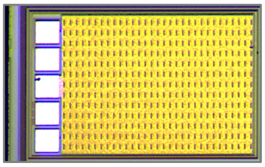	IDC implemented in Metal 3<br>Pads in Metal 4 (top metal)<br>Shield implemented using thick Metal 4 (2.8 μm height)<br>Bond pads size: 150 x 150 μm^2<br>Passivated everywhere except pads<br>WoV around the whole structure<br>Finger width = 1 μm<br>Finger spacing = 0.6 μm<br>Number of fingers = 606 (303 each electrode)<br>Finger length = 664 μm

*The two test structures on Chip-A were fabricated adjacent to other circuity intended for a different project which was not used in this investigation.

Table S2: Chip-B test structures with specifications: interdigitated capacitor (IDC) and negative metal oxide semiconductor (NMOS) test structures implemented using a 0.18 μm 6-metal process.

Structure	Circuit layout	Specifications
**M5-IDC**<br>(1.1 mm x 0.7 mm)	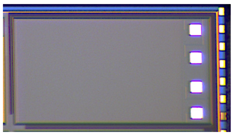	IDC implemented in Metal 5<br>Pads in Metal 6<br>Bond pads size: 80 x 80 μm^2<br>Passivated everywhere except pads<br>Wall of via (WoV) around the whole structure<br>Finger width = 1 μm<br>Finger gap = 0.6 μm<br>Number of fingers = 716 (358 each electrode)<br>Finger length = 700 μm
**M6-SH/M5-IDC**<br>(0.9 mm x 0.6 mm)	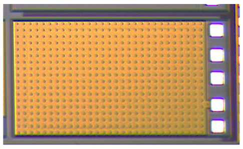	IDC implemented in Metal 5<br>Pads in Metal 6<br>Shield implemented using Metal 6 (1 μm height)<br>Bond pads size: 80 x 80 μm^2<br>Passivated everywhere except pads<br>Wall of via (WoV) around the whole structure<br>Finger width = 1 μm<br>Finger gap = 0.6 μm<br>Number of fingers = 606 (303 each electrode)<br>Finger length = 609 μm
**M6-IDC**<br>(1.1 mm x 0.7 mm)	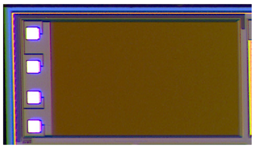	IDC implemented in Metal 6<br>Pads in Metal 6 (top metal)<br>Bond pads size: 80 x 80 μm^2<br>Passivated everywhere except pads<br>Wall of via (WoV) around the whole structure<br>Finger width = 1 μm<br>Finger gap = 1 μm<br>Number of fingers = 716 (358 each electrode)<br>Finger length = 704 μm
**NMOS**	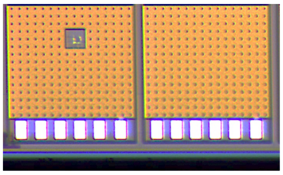	NMOS transistors, with and without shield.<br>Pads in Metal 6 (top metal)<br>Shield in Metal 5 and 6 (double shielded)<br>Passivated everywhere except pads<br>NMOS total W = 400 μm (W = 20 μm with N = 20 fingers)<br>NMOS L = 0.36 μm<br>Electro static protection used for all pads

Table S3: Overview of test structures used in various aging environments, along with the electrical and material analysis tools applied to each structure. Values in red give the month at which a sample failed. In the table below, 'n' gives the number of samples which was used for aging tests or post-aging analysis.

Chip	Test structure	Aging	DC bias	Months in test (total number of tested samples)	Electrical[2]	AFM/ SEM[3]	ToF-SIMS/ XPS[4]
A (n=28)	M3-IDC	PBS @ 67 °C	unbiased	6, 7, 10, 12, 16 (n=5)	EIS	6-month (n=2) 10-month (n=2)	7-month (n=1) 12-month (n=2)
			5 V, IDC	12 (n=2)	EIS	12-month (n=1)	12-month (n=1)
			5 V, IDC(-) to PBS(+)	12 (n=2)	EIS	12-month (n=2)	12-month (n=2)
			15 V, IDC	3, 12, 16 (n=4)	EIS	12-month (n=1)	12-month (n=1)
			15 V, IDC(-) to PBS(+)	12 (n=2)	EIS	12-month (n=1)	12-month (n=2)
		DI @ 67 °C	15 V, IDC	5, 16 (n=2)	EIS	12-month (n=1)	12-month (n=1)
		rat[5]	Unbiased	3, 7,12 (n=5)	-	7-month (n=2) 12-month (n=2)	7-month (n=2) 12-month (n=2)
	M4-SH/ M3-IDC	PBS @ 67 °C	15 V, IDC	5, 12 (n=4)	EIS	12-month (n=1)	-
			5V, M4(-) to PBS(+)	7 (n=4)	EIS	7-month (n=3)	-
B (n=48)	M5-IDC	PBS @ 67 °C	unbiased	6, 12, 16 (n=4)	EIS	6-month (n=2) 12-month (n=2)	7-month (n=2) 12-month (n=2)
			5 V, IDC	12 (n=4)	EIS	-	-
			5 V IDC(-) to PBS(+)	12 (n=2)	EIS	12-month (n=1)	12-month (n=2)
			15 V, IDC	12 (n=3)	EIS	12-month (n=1)	12-month (n=1)
			15 V, IDC(-) to PBS(+)	12, 16 (n=3)	EIS	12-month (n=1)	12-month (n=2)
		DI @ 67 °C	15 V, IDC	16 (n=2)	EIS	16-month (n=1)	16-month (n=1)
		rat	unbiased	3, 7,12 (n=5)	-	7-month (n=2) 12-month (n=2)	7-month (n=2) 12-month (n=2)
	M6-IDC	PBS @ 67 °C	unbiased	3, 16 (n=4)	EIS	12-month (n=1)	-
			5 V, IDC	1, 3, 12 (n=5)	EIS	12-month (n=2)	-
			15 V, IDC	1, 12, 16 (n=5)	EIS	12-month (n=2)	-
	M6-SH/ M5-IDC	PBS @ 67 °C	5 V, IDC	10, 12 (n=5)	EIS	12-month (n=1)	-
	MOS	PBS @ 67 °C	unbiased	12 (n=4)	V_{GS} - I_{DS}	12-month (n=2)	-
	M6-M5-SH/ MOS	PBS @ 67 °C	unbiased	12 (n=1)	V_{GS} - I_{DS}	-	-
	Dielectric sensor	PBS @ 67 °C	unbiased	5 (n=1)	array measurements	-	-

Optical microscopy (up to 200x magnification) was done on all devices.

[1]For some test structures, samples were taken out at months 3, 6 or 10 for material analysis.

[2]All electrical measurements were done monthly until the end of the *in vitro* accelerated aging.

[3]For samples analyzed using both ToF-SIMS/XPS and SEM, the SEM analysis was performed after the ToF-SIMS/XPS analysis, as the sample surface must be coated with a thin conductive Pt layer for SEM. Therefore, all surface analysis techniques, such as AFM, ToF-SIMS, and XPS, were conducted prior to SEM.

[4]ICs were first analyzed using ToF-SIMS, both in positive and negative modes. For quantification, ICs were further analyzed using XPS. During the ToF-SIMS analysis of aged ICs, reference ICs (pristine, as is from foundry) were also used for comparison.

[5]In vivo samples were first microscopically inspected, decapsulated and again inspected using microscopy. AFM at the PDMS-edge boundary was used to determine the thickness of the SiN_X/SiO_X passivation. Later, samples were analyzed using ToF-SIMS and XPS. Finally, the ICs were inspected with SEM.

Table S4: Properties of the two PDMS materials used during the long-term aging studies (data obtained from the manufacturer datasheet).

	**Dow Corning 3140**	**NuSil MED2-4213**
**Aging study**	Accelerated aging	In vivo anmial
**Medical grade**	No	Yes
**Processing**	Dispensing	Dispensing
**Cure System**	1-part acetoxy	2-part heat addion cure
**Mix ratio**	-	1:1
**Cure time and temperature**	3 days @ 25 °C	2 hours @ 100 °C
**Adhesive**	Yes	Yes
**Youngs's modulus (MPa)**	0.7	0.6
**Tensile strength (MPa)**	3	6.9
**Durometer shore A**	31	15
**Uncured viscocity (mPa*s)**	34400	80000
**Volume resitivity (Ohms*cm)**	2.1 x 10^{14}	-
**Dielectric strengh kV/mm**	15	-

For this investigation, the key material properties of the two selected PDMS elastomers were: 1) softness (Durometer-A), 2) strong and long-lasting underwater adhesion to silicon-based ceramics, and 3) a low-temperature curing system. The lower temperature curing system minimizes the interfacial stresses created with PDMS. The long-lasting adhesion properties of the two PDMS elastomers were already demonstrated in our prior experiments with these materials.

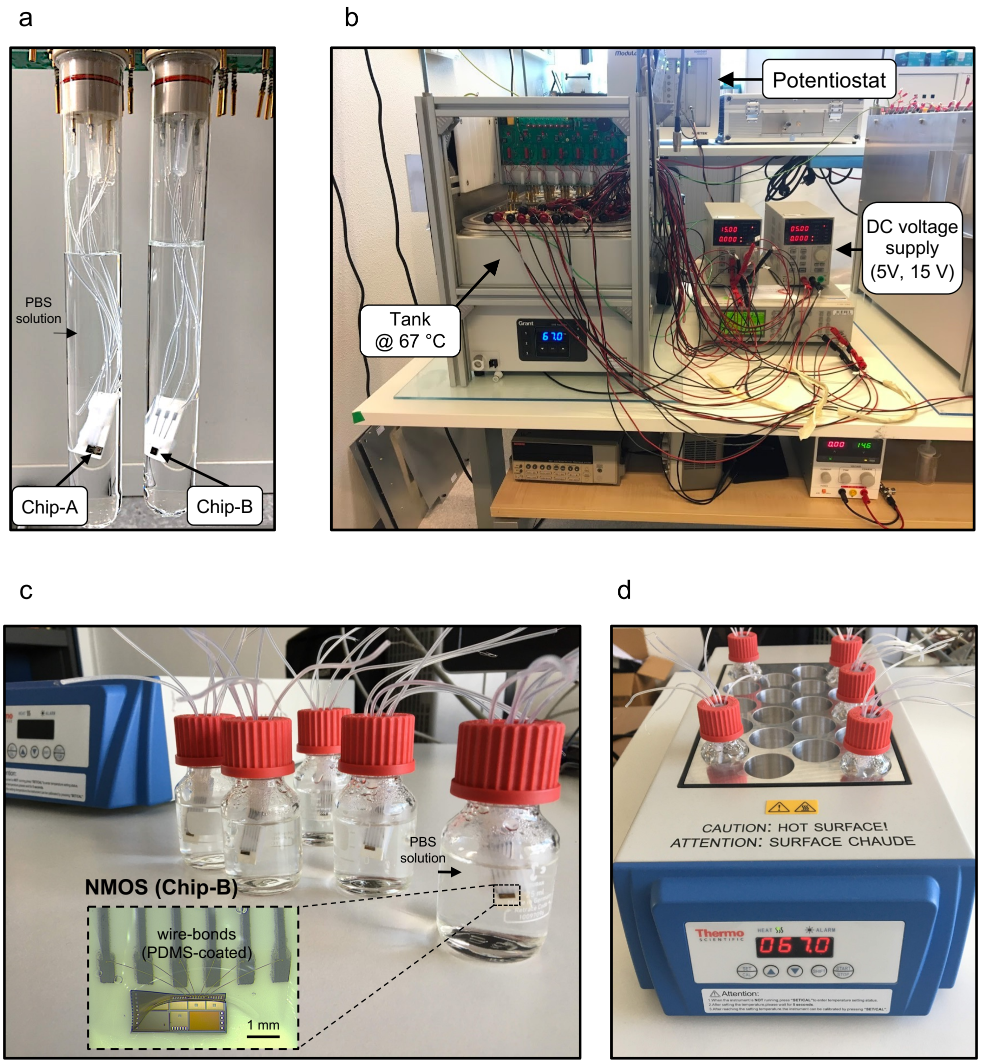

Figure S5. Set-ups used for the accelerated *in vitro* aging studies. **a-b)** Set-up used for aging the interdigitated capacitor (IDC) structures. **a)** Chip-A and B ICs connected to 3-contact ceramic (alumina) adaptors while immersed in vials filled with 50 ml of phosphate buffered saline (PBS) solution. **b)** Heat-regulated water-bath tank and voltage supplies (5 V and 15 V DC) used for accelerated aging and electrical stressing of IDC test structures. Potentiostat used for electrochemical impedance spectroscopy (EIS). More information on the set-up can be found in [1]. **c)** N-channel metal oxide semiconductor (NMOS) transistors (Chip-B) wire-bonded to 6-contact ceramic substrates (inset), partially PDMS-coated and immersed in PBS solution, **d)** heater used to maintain a constant temperature at 67 °C.

a

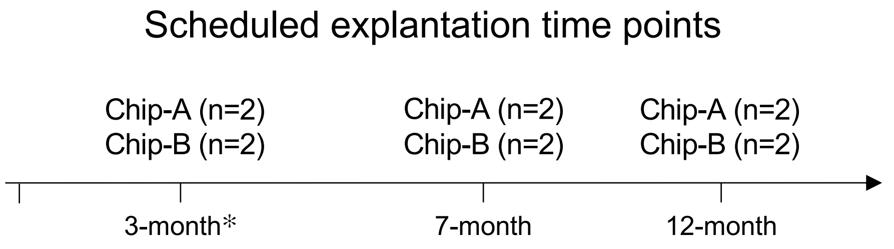

b

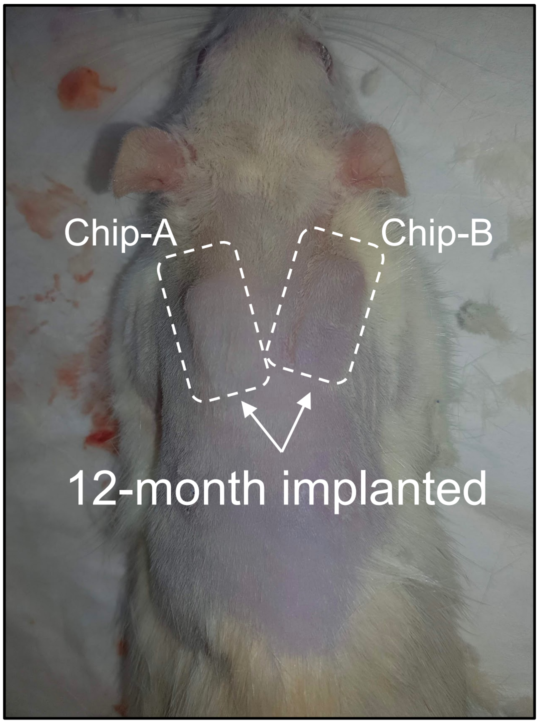

Figure S6. **a)** Explantation time points for the implanted Chip-A and B ICs in rats. **b)** image of a rat animal model after 12 months of implantation with complete wound healing and no observable inflammation around the two implanted chips (* One animal died during the study due and was incinerated with the device still implanted). 'n' gives the number of samples which were explanted at specific time points (3, 7 and 12 months).

Chemical composition of IC passivation layers (as received from foundry)

Table S5: Chemical composition of the SiN_X and SiO_X passivation layers for Chip-A and B ICs (as received from foundry), determined using X-ray photoelectron spectroscopy (XPS) surface and depth profiling analysis. The measured carbon (C) is from surface contamination and is generally found at the surface of samples exposed to ambient air. After 1 sputter cycle the carbon contamination is removed.

Chip A	Measured Depth (nm)	Si (at%)	N (at%)	O (at%)	C (at%)
SiN_X	0[a]	29.9	7.9	39.8	21.8[b]
	5	49.7	45.5	4.8	0
	10	51.5	48.5	0	0
	14	50.6	49.4	0	0
SiO_X	~1050	33	0	67	0
**Chip B**	**Measured Depth (nm)**	**Si (at%)**	**N (at%)**	**O (at%)**	**C (at%)**
SiN_X	0[a]	26.7	10.8	36.1	26.3
	5	49.6	47	3.5	0.1
	10	49.4	49.3	1.3	0
	14	49.0	51	0	0
SiO_X	~1100	32.7	0	67.3	0

[a] For XPS surface measurement, the information depth is approximately 7 nm.
[b] The carbon detected on the surface is from ambient environment (adventitious carbon).

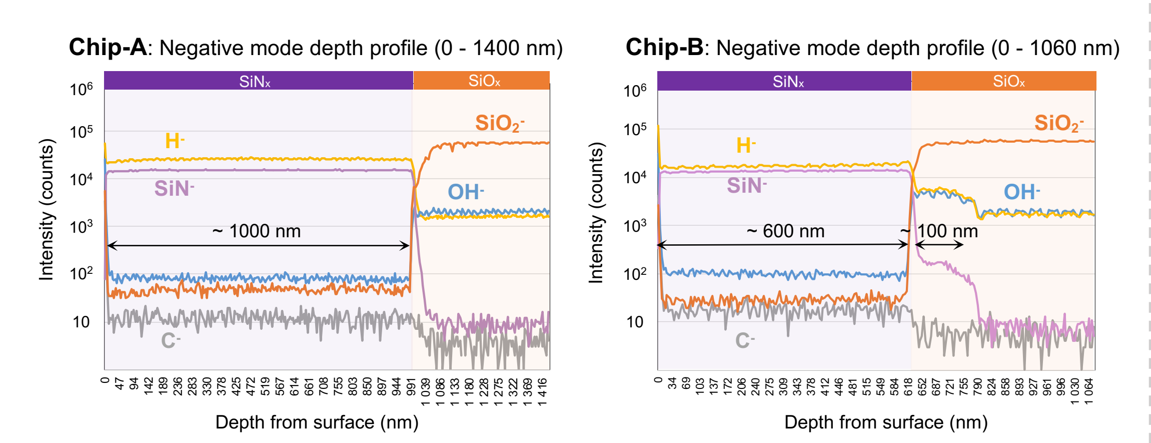

Figure S7. Negative mode time-of-flight mass spectrometry (ToF-SIMS) depth profiles of Chip-A (right) and Chip-B (left) ICs (as received from foundry).

The quantification of the hydrogen content (H) in the SiN_X passivation was done in ToF-SIMS software using a relative sensitivity factor (RSF). The RSF was derived from measurement results performed on known SiN:H reference layers. The hydrogen content on these SiN:H reference layers has been previously calibrated using elastic recoil detection (ERD).

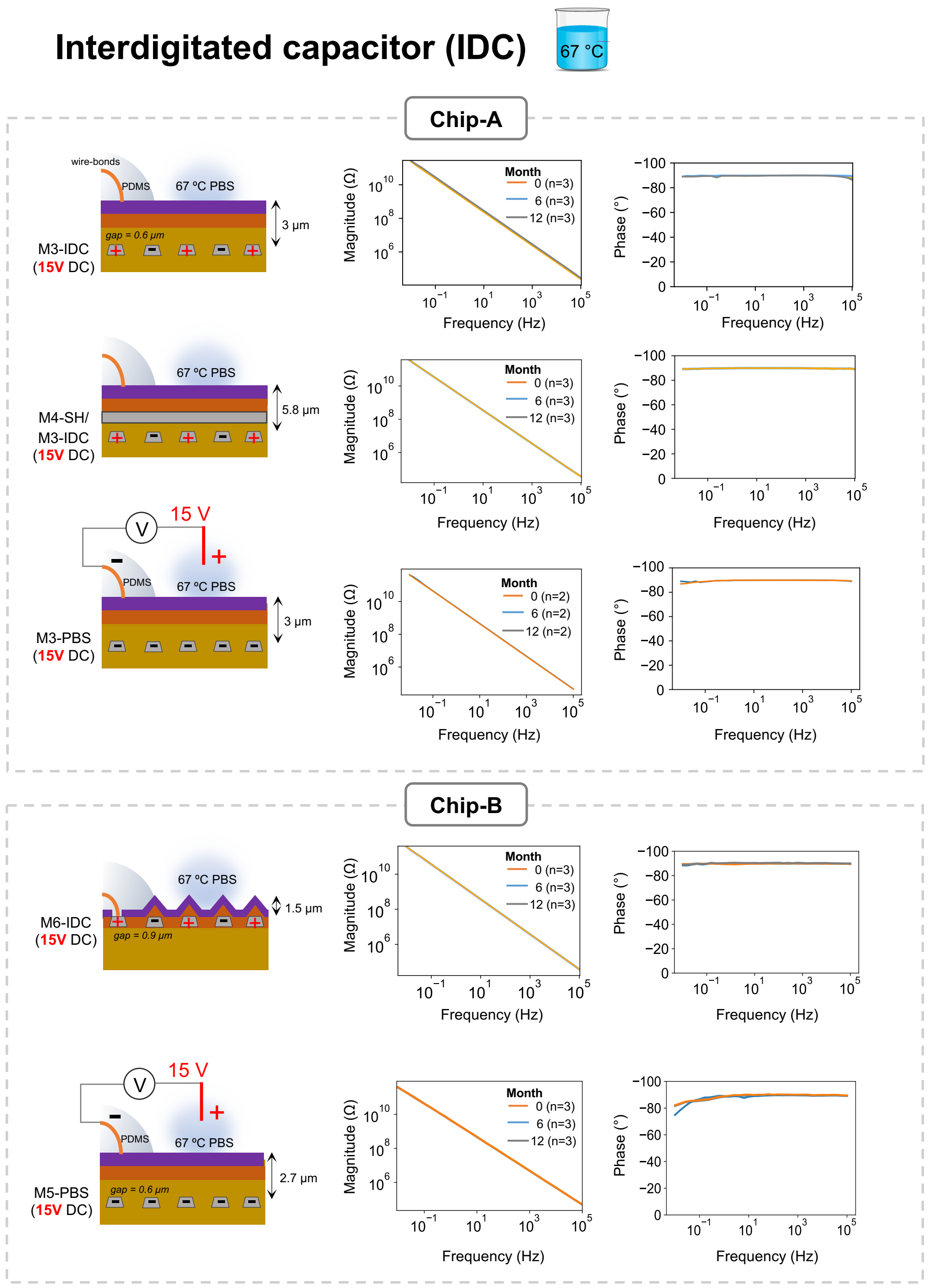

Figure S8. Electrochemical impedance spectroscopy (EIS) results of interdigitated capacitor (IDC) test structures on Chip-A and Chip-B ICs over the 12-month accelerated *in vitro* aging in PBS solution at 67 °C. Schematics show the distance between the metal test structures within the IC and the surface which is exposed to PBS solution (dimensions not to scale). Values are presented as the average of ‘n’ samples, where ‘n’ represents the number of samples used for each test structure at each time point.

Electrical failures

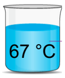

Table S6: Overview of test structures and the failures observed during the accelerated *in vitro* aging study. In the table below, 'n' gives the number of samples which was used for aging tests. Values in red give the time point (month) and sample size which failed.

Chip	Test structure	Aging	DC bias	Months in test (total number of tested samples)	Time to failure (number of failed samples)	Failure
A (n=28)	M3-IDC	PBS @ 67 °C	unbiased	6, 7, 10, 12, 16 (n=5)	7-month (n=1)	Wire-bond corrosion
			5 V, IDC	12 (n=2)	-	-
			15 V, IDC	3, 12, 16 (n=4)	3-month (n=1)	Wire-bond corrosion
			15 V, IDC(-) to PBS(+)	12 (n=2)	-	-
		DI @ 67 °C	15 V, IDC	5, 16 (n=2)	5-month (n=1)	Wire-bond corrosion
		rat	Unbiased	3, 7,12 (n=5)	-	-
	M4-SH/ M3-IDC	PBS @ 67 °C	15 V, IDC	5, 12 (n=4)	5-month (n=1)	Wire-bond corrosion
			5V, M4(-) to PBS(+)	7 (n=4)	7-month (n=3)	IC passivation crack/opening due to use of top metal
B (n=48)	M5-IDC	PBS @ 67 °C	unbiased	6, 12, 16 (n=4)	-	-
			5 V, IDC	12 (n=4)	-	-
			5 V IDC(-) to PBS(+)	12 (n=2)	-	-
			15 V, IDC	12 (n=3)	-	-
			15 V, IDC(-) to PBS(+)	12, 16 (n=3)	-	-
		DI @ 67 °C	15 V, IDC	16 (n=2)	-	-
		rat	unbiased	3, 7,12 (n=5)	-	-
	M6-IDC	PBS @ 67 °C	unbiased	3, 16 (n=4)	3-month (n=2)	IC passivation crack/opening due to use of top metal
			5 V, IDC	1, 3, 12 (n=5)	1-month (n=1)<br>3-month (n=1)	IC passivation crack/opening due to use of top metal
			15 V, IDC	1, 12, 16 (n=5)	1-month (n=2)	IC passivation crack/opening due to use of top metal
	M6-SH/ M5-IDC	PBS @ 67 °C	5 V, IDC	10, 12 (n=5)	10-month (n=1)	Wire-bond corrosion
	MOS	PBS @ 67 °C	unbiased	12 (n=4)	-	-
	M6-M5-SH/ MOS	PBS @ 67 °C	unbiased	12 (n=1)	-	-
	Dielectric sensor	PBS @ 67 °C	unbiased	5 (n=1)	5-month (n=1)	Wire-bond corrosion

During the accelerated *in vitro* aging study, a group of samples showed irregularities in the electrical results. Table S6 gives an overview of these test structures. These samples were taken out from the remainder of the accelerated study and were analyzed using optical and electron microscopy. Results showed failures to be either due to wire-bond corrosion or due to the use of the top metal layer which resulted in stress-induced cracks/openings in the passivation or, in the poor conformality of the layer.

Wire-bond corrosion

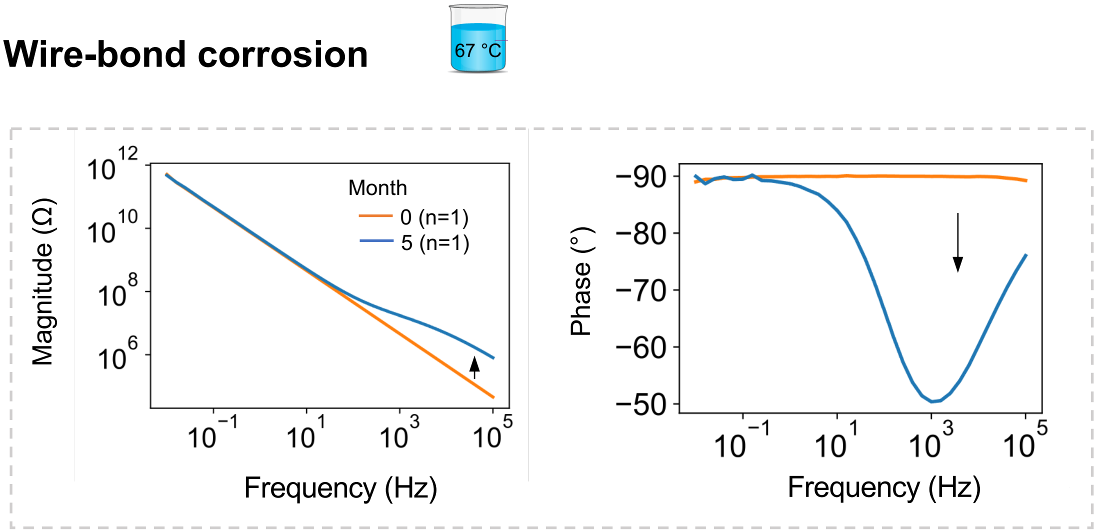

After PDMS decapsulation

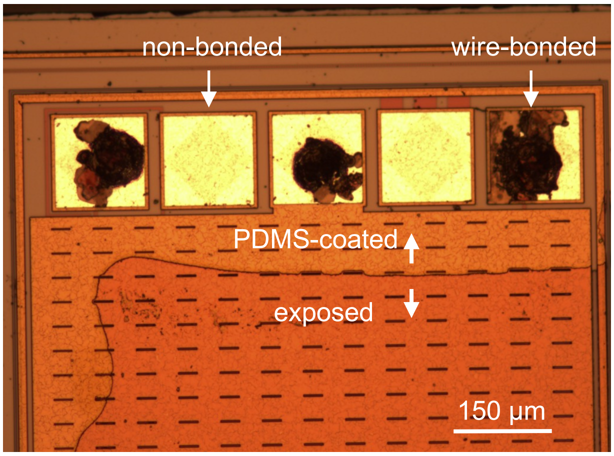

Figure S9. Electrochemical impedance spectrometry (EIS) results and optical micrographs of sample with wire-bond corrosion in the PDMS-coated region after 5-months of soaking in PBS solution at 67°C and applied to 15 V DC bias. EIS results presented as Bode plots for a M4-SH/M3-IDC test structure (Chip-A) at 5-month show change in higher frequency ranges. 'n' gives the sample size used for measurement. Optical micrograph of the structure after PDMS decapsulation show aluminum corrosion on the wire-bonded pads. No corrosion is seen for the non-bonded pads.

In this study, a number of IDC samples (n=5 out of in total n=56) showed change in EIS results in the frequency range of 10 Hz to 100 KHz. Optical microscopy revealed that these samples experienced wire-bond corrosion which increased the ohmic value of the interconnect joint. Closer examination revealed the corrosion to only occur on pads connected to wire-bonds (Figure S9). For PDMS-coated wire-bonds, this type of corrosion has been reported before [2-3] and is most likely due to the galvanic corrosion at the Al-Au interface which is triggered by moisture. The galvanic corrosion results in the degradation of the less noble metal, in this case, aluminum (Al). PDMS-coated aluminum pads without gold (Au) wire bonds, on the other hand, did not exhibit any corrosion on all tested samples during the accelerated study in PBS solution at 67 °C. This is mainly due to the strong adhesion between the PDMS and aluminum which has also been demonstrated in previous studies [2].

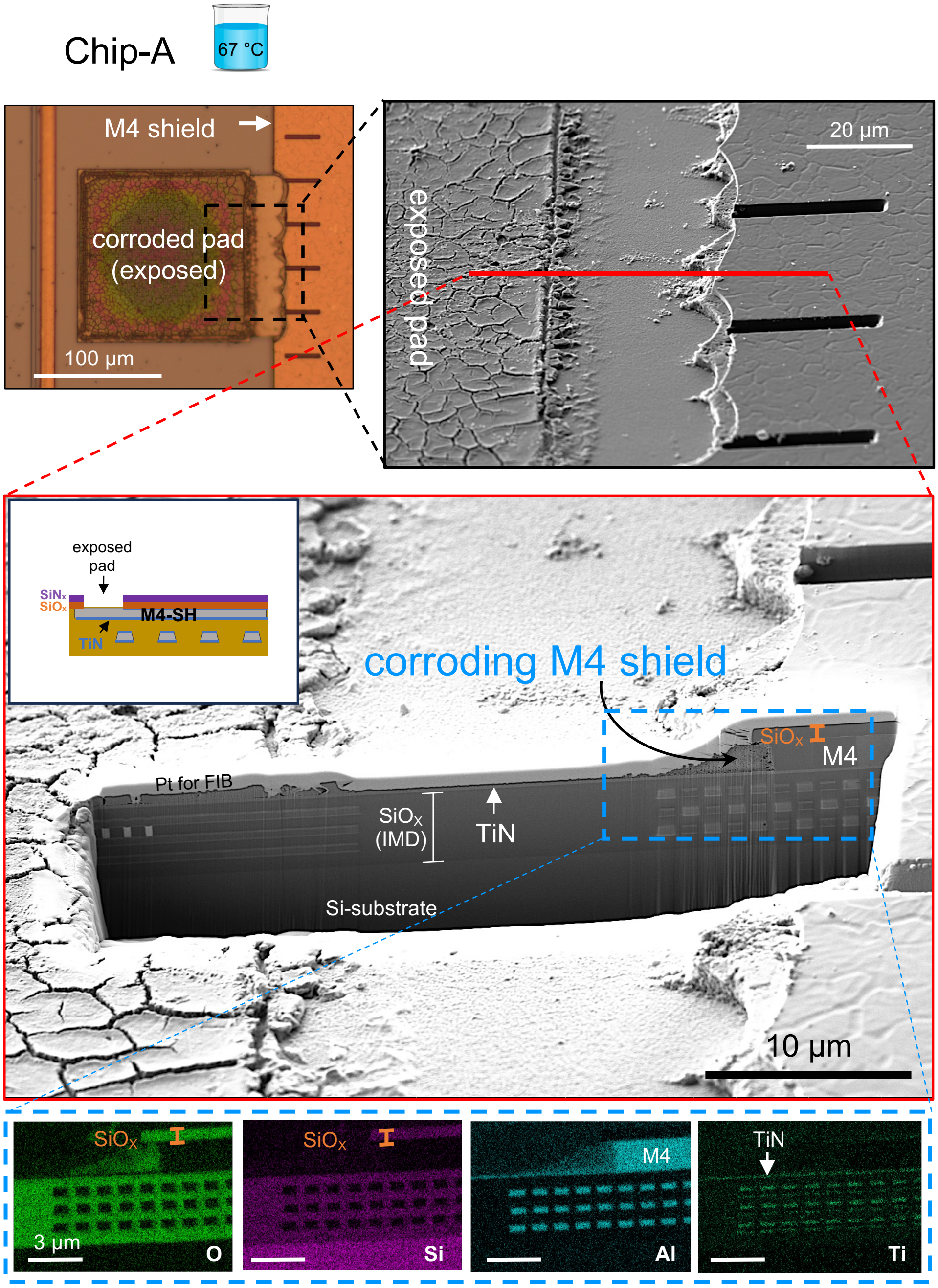

Figure S10. Optical and electron micrographs and EDX elemental mapping of an exposed aluminum pad connected to top metal shield (M4-SH) on a Chip-A sample after 10 months immersion in PBS solution at 67 °C. Red line indicates the FIB cut for cross section analysis. Scale bar for all EDX maps are similar (3 µm).

Metal pads are openings in the passivation to allow for electrical connections to the chip. Due to the opening, we were concerned if the corrosion of the aluminum pads would allow any liquid ingress, specially from the edges of the pads (the boundary between metal and the passivation). For this purpose, during the accelerated *in vitro* aging, to evaluate the worst-case scenario some pads were intentionally left uncoated and exposed to PBS solution. Figure S10 shows an exposed pad connected to the M4-SH layer. After 10 months of soaking, severe corrosion was observed. Despite severe corrosion, cross sectional SEM imagining and EDX elemental mapping revealed intact buried Al metallization. The thin titanium nitride (TiN) layer, which is used by the foundries as a metal diffusion barrier [4], is also visible with no signs of corrosion, demonstrating its high stability in PBS solution at 67 °C. Results indicate no ingress of corrosive liquid into the chip from the pad openings.

Passivation planarity and stress-induced cracks

Supplementary Note 4: In the fabrication process of silicon ICs, each metal layer is covered by an insulating layer. For the deep metal layers, i.e., M1-M3 or M1-M5, for Chip-A and Chip-B, respectively, this insulating layer is the SiO_X intermetallic dielectric (IMD) layer and is always followed by a planarization step. When the topmost metal is used, i.e., M4 or M6, for Chip-A and Chip-B, respectively, the IC's passivation (PECVD layer of SiO_X followed by SiN_X) is directly deposited on top with no final planarization step. In this case, the microtopography of that topmost metal layer will define the planarity of the IC's passivation (see Figures S2 and S3). A non-planar passivation layer may experience stress at the high-aspect ratio features resulting in cracks [5]. Such stress-induced cracking would greatly compromise the barrier performance of the passivation and allow direct ingress of water/ions within the IC.

In our study, such stresses was shown to be a source of failure in test structures where the topmost metal was included in the IDC design. In fact, Figures S11 and S12 depict the observed passivation cracking and subsequent metal corrosion which was introduced or accelerated while electrically biasing the topmost metal, both for Chip-A and B. None of the IDC structures implemented with lower metallization exhibited such failures.

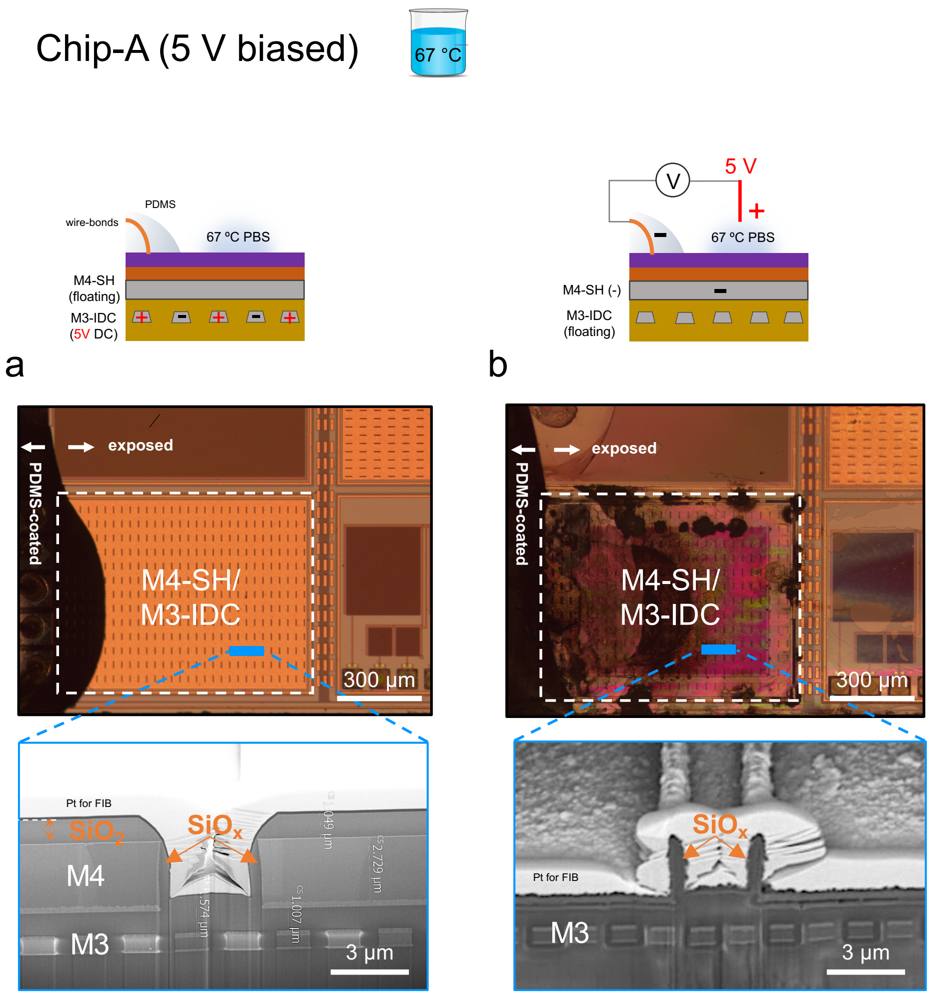

Figure S11. Optical micrograph and cross-sectional SEM images of two M4-SH/M3-IDC test structures (Chip-A) after accelerated *in vitro* testing with biasing. **a**) Optical micrograph of a sample after 10 months of accelerated aging at 67 °C with a continuous 5 V bias applied between the combs of the M3-IDC. M4-SH was not biased and was left floating. Blue line indicates FIB cut for cross section analysis. Cross-sectional SEM image showing total dissolution of the SiN_X passivation after 10 months of soaking, nearly exposing the edges of the M4 metal to the PBS solution. **b**) Optical micrograph of a different Chip-A sample with a similar test structure. After 9 months of 5 V DC bias between the M4-SH metal and PBS solution severe degradation is visible. Cross-sectional SEM image of the damaged area shows complete loss of both passivation layers (SiN_X and SiO_X) and the M4-SH (2.8 µm) metal layer, leaving only the SiO_X edge side walls. The M3-IDC and other buried material stacks remained intact despite the aggressive electrolysis due to the DC biasing.

Utilizing the top-most metal results in microtopography on the IC surface. Figure S11 shows the M4-SH/M3-IDC test structure on two different Chip-A samples after accelerated *in vitro* testing. On these test structures, the presence of slots in the top shield (M4-SH) metal layer creates microtopography on the IC's surface. This microtopography results in poor conformality and increased stress in the IC's passivation layers [4], leading to earlier-than-expected exposure of the IDC metals to the PBS solution. When the metals are exposed and applied to a DC electrical voltage, severe degradation is observed as a result of water electrolysis and gas evolution (H_2 and O_2) [3].

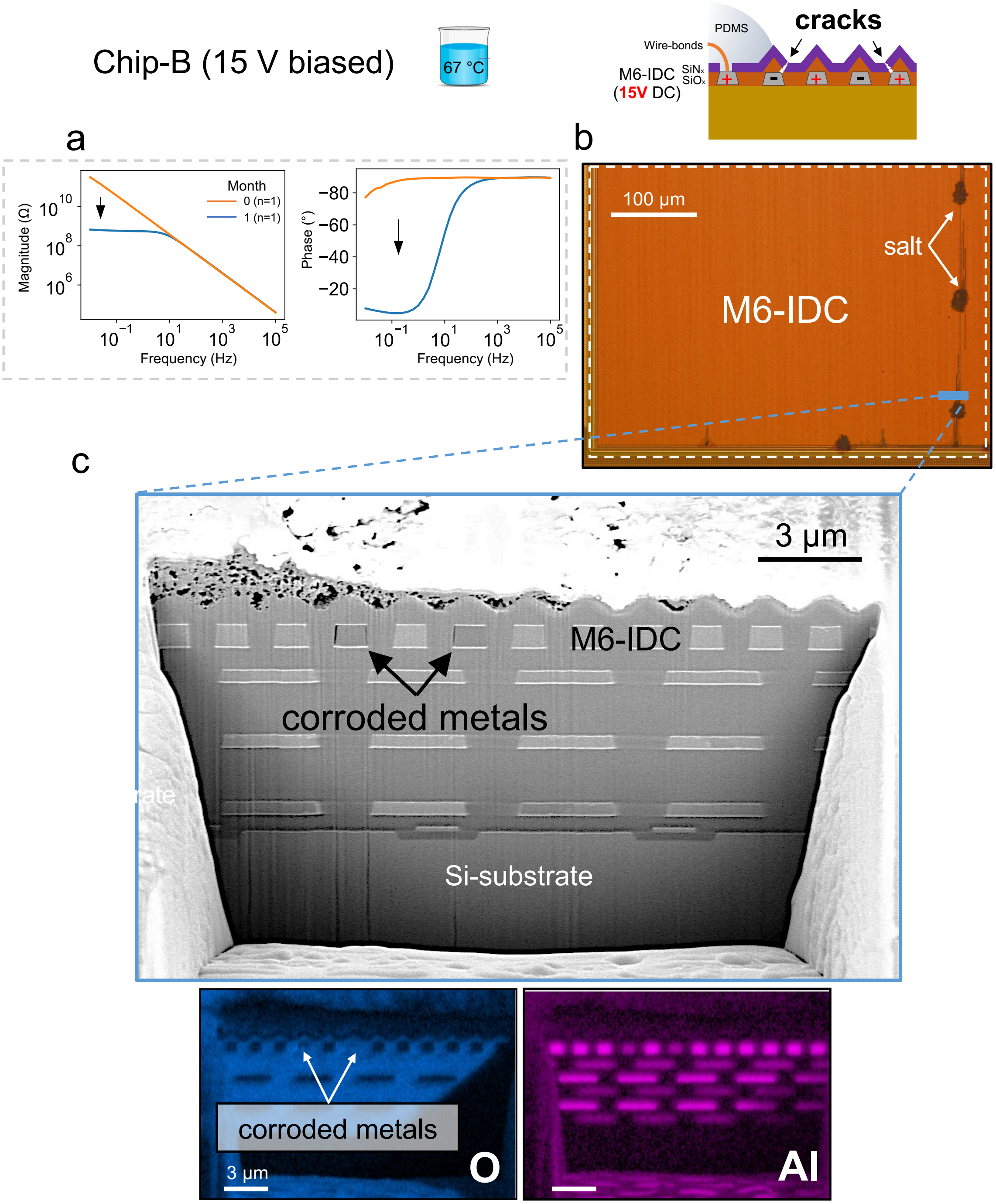

Figure S12. Failure on a 15V DC biased M6-IDC test structure. **a**) EIS results given as Bode plots for a representative M6-IDC (Chip-B) structure showing EIS irregularities after being applied to a 15 V DC bias voltage for 1-month. EIS results at 1-month show a significant drop in magnitude with more resistive behavior (phase ~ -20) at frequencies below 10 Hz. 'n' gives the sample size used for measurement. **b)** Optical micrograph of the representative M6-IDC structure with noticeable metal corrosion and salt residue on the corroded sites. Blue line indicates the FIB cut used for cross section analysis. **c)** Cross-sectional SEM image and EDX elemental mapping showing higher oxygen content for the corroded aluminum fingers. Scale bar for EDX maps are all similar (3 µm).

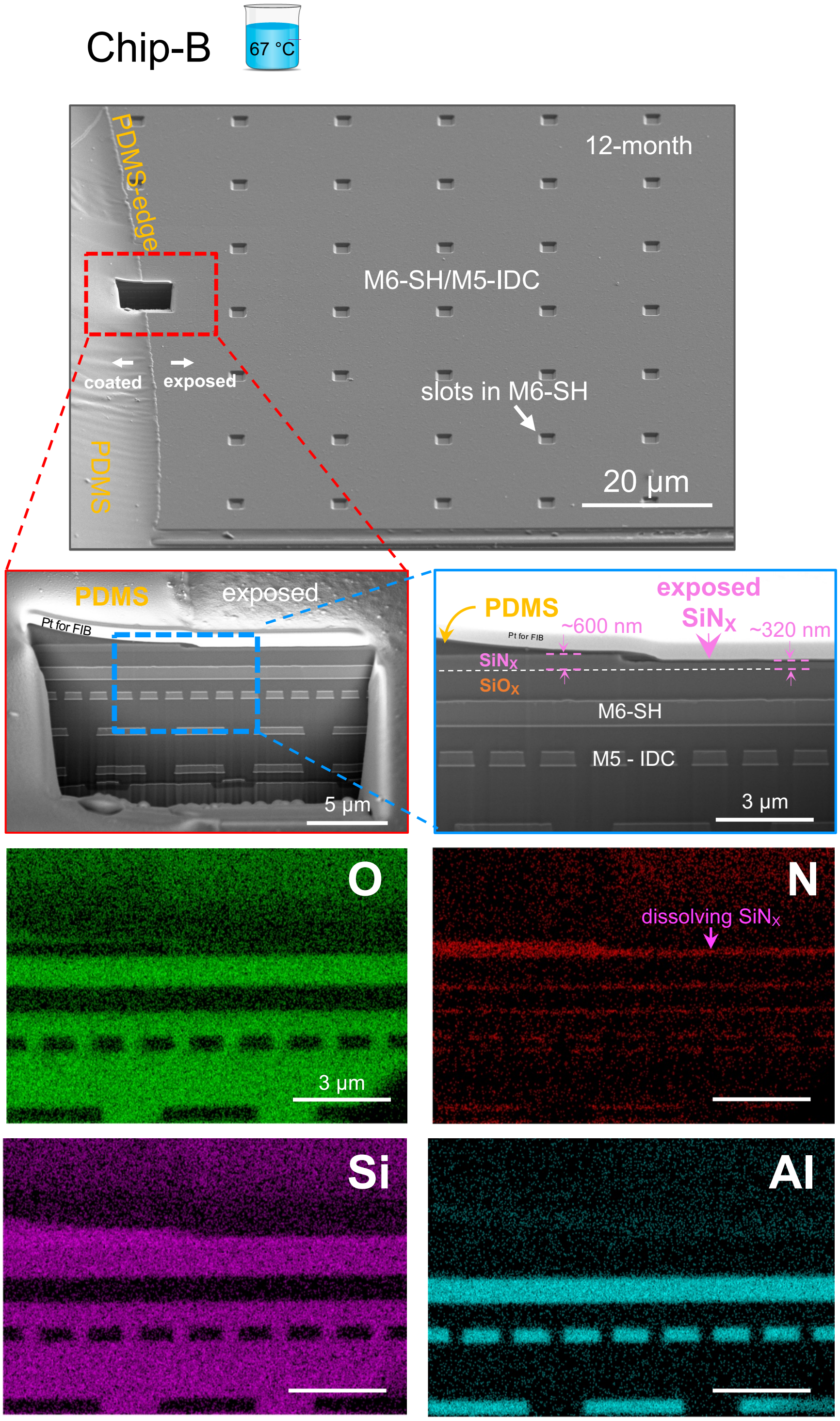

Figure S13. Tilted SEM surface image of a M6-SH/M5-IDC structure (Chip-B) after 12 months accelerated aging in PBS solution at 67 °C. Slots in M6 shield barrier result in microtopography on the IC surface. Slots are created to obey the metal density rules specified by the IC foundry. FIB cut (red square) on the PDMS-edge is used to evaluate the IC multilayer stack stability. Similar cuts were done on two other locations on the chip. Magnified SEM images and EDX elemental mapping of cross-section reveals a dissolution of the SiN_X passivation in the exposed area, leaving ~320 nm of SiN_X in the exposed region of the IC. This results in a dissolution rate of ~ 23 nm/month for the SiN_X on Chip-B in PBS solution at 67 °C. The PDMS-coated area appears intact (~600 nm). Scale bar for EDX maps are all similar (3 µm).

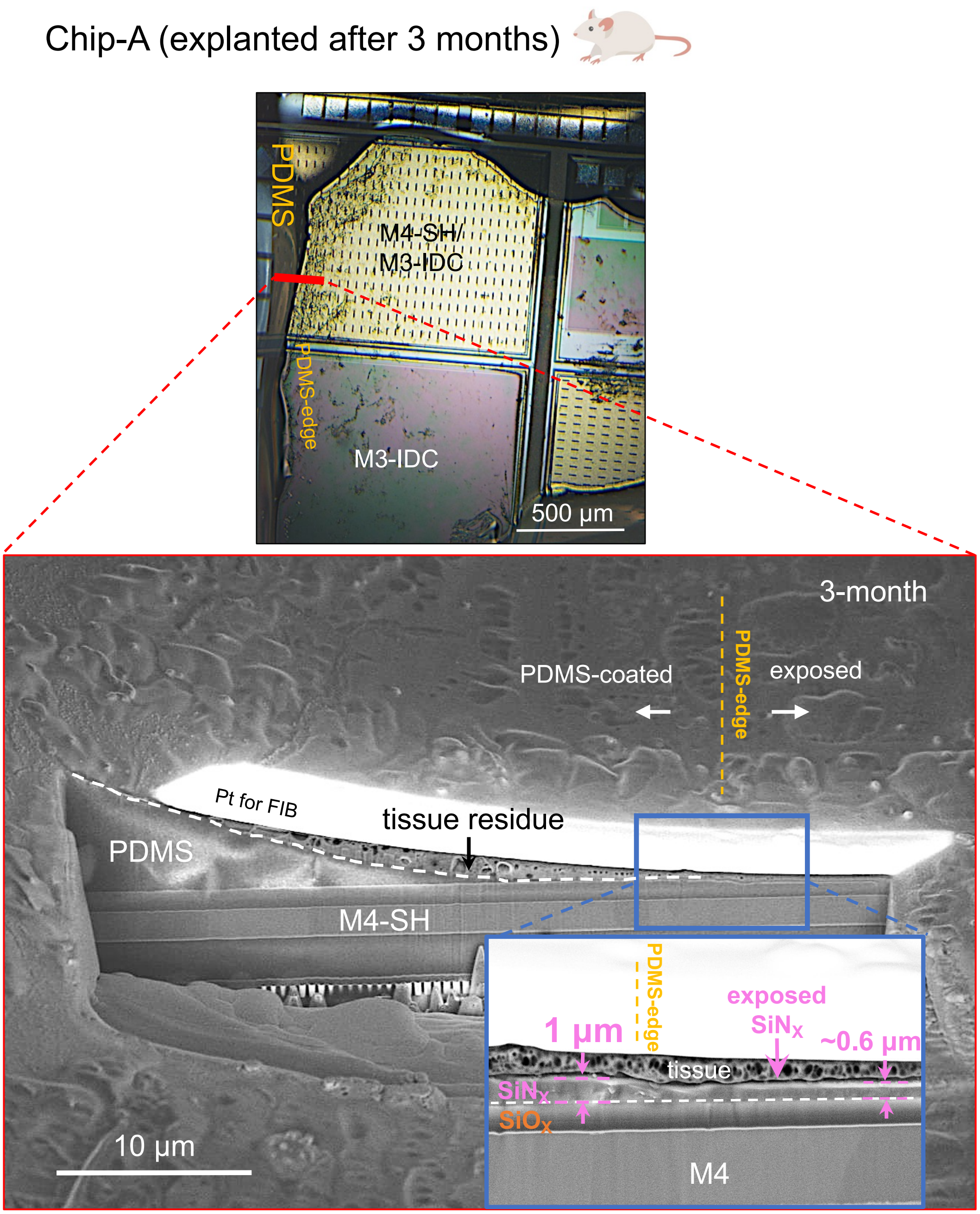

Figure S14. Optical micrograph of a Chip-A sample explanted after 3 months in rat (before cleaning and tissue removal) (top). Red line indicates FIB cut used for cross section analysis. Cross sectional SEM image near the PDMS-edge of the M4-SH/M3-IDC test structure showing the IC material stack (bottom). Inset: magnified SEM image of the cross-section near the PDMS-edge, comparing the top SiN_X and SiO_X passivation layers in the PDMS-coated and exposed (uncoated) regions. Dissolution of the SiN_X passivation in the exposed region is visible, while no dissolution is observed in the PDMS-coated region. The thickness of the PDMS coating near the PDMS-edge is less than 1 µm. At this region, the thin PDMS is still preventing the SiN_X passivation from tissue contact and dissolution. The remaining IC material stack (intermetallic dielectric and metallization) show no signs of delamination or degradation and remain intact.

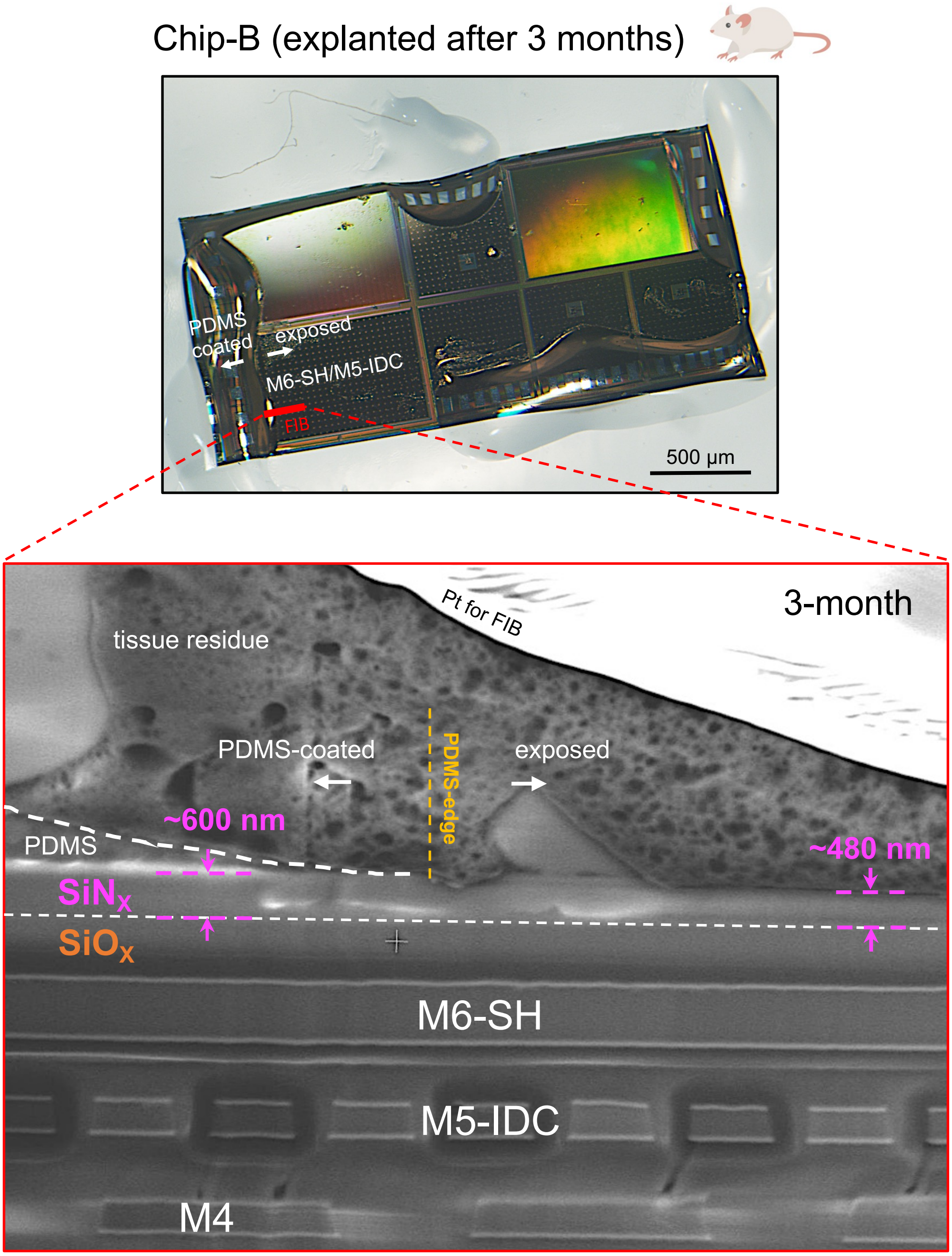

Figure S15. Optical micrograph of an explanted Chip-B sample after 3 months exposure to body environment (before cleaning and tissue removal). Red line indicates FIB cut used for cross section analysis. SEM cross-sectional image on the PDMS-edge of M6-SH/M5-IDC test structure show visible dissolution of SiN_X passivation in the uncoated region. The remaining IC material stack (intermetallic dielectric and metallization layers) show no signs of delamination or degradation and remain intact. Note that the thickness of the PDMS coating near the PDMS-edge is less than 1 µm and is still protecting the SiN_X passivation from tissue contact and dissolution.

Supplementary Note 5: The 7-month and 12-month explanted samples were analyzed using optical microscopy and AFM. Samples were first cleaned from tissue residues, PDMS decapsulated and later analyzed using optical microscopy and AFM on a 20 µm x 20 µm area at the PDMS-edge (**Figure S16** and **S17**). Optical inspections revealed a non-uniform color on the exposed regions of the chip surface, indicating possible non-uniform dissolution of the passivation. The non-uniform dissolution could be due to the non-homogeneous coverage of various enzymes and tissue on the IC's surface. After decapsulation, PDMS protected regions were similarly inspected, showing no signs of degradation (See **Figure S16**). Additionally, all the aluminum pads also remained intact due to the excellent adhesion of PDMS to aluminum. AFM analysis on the 7-month and 12-month explanted ICs showed a loss of the SiN_X passivation (**Figure S17** and **S18**).

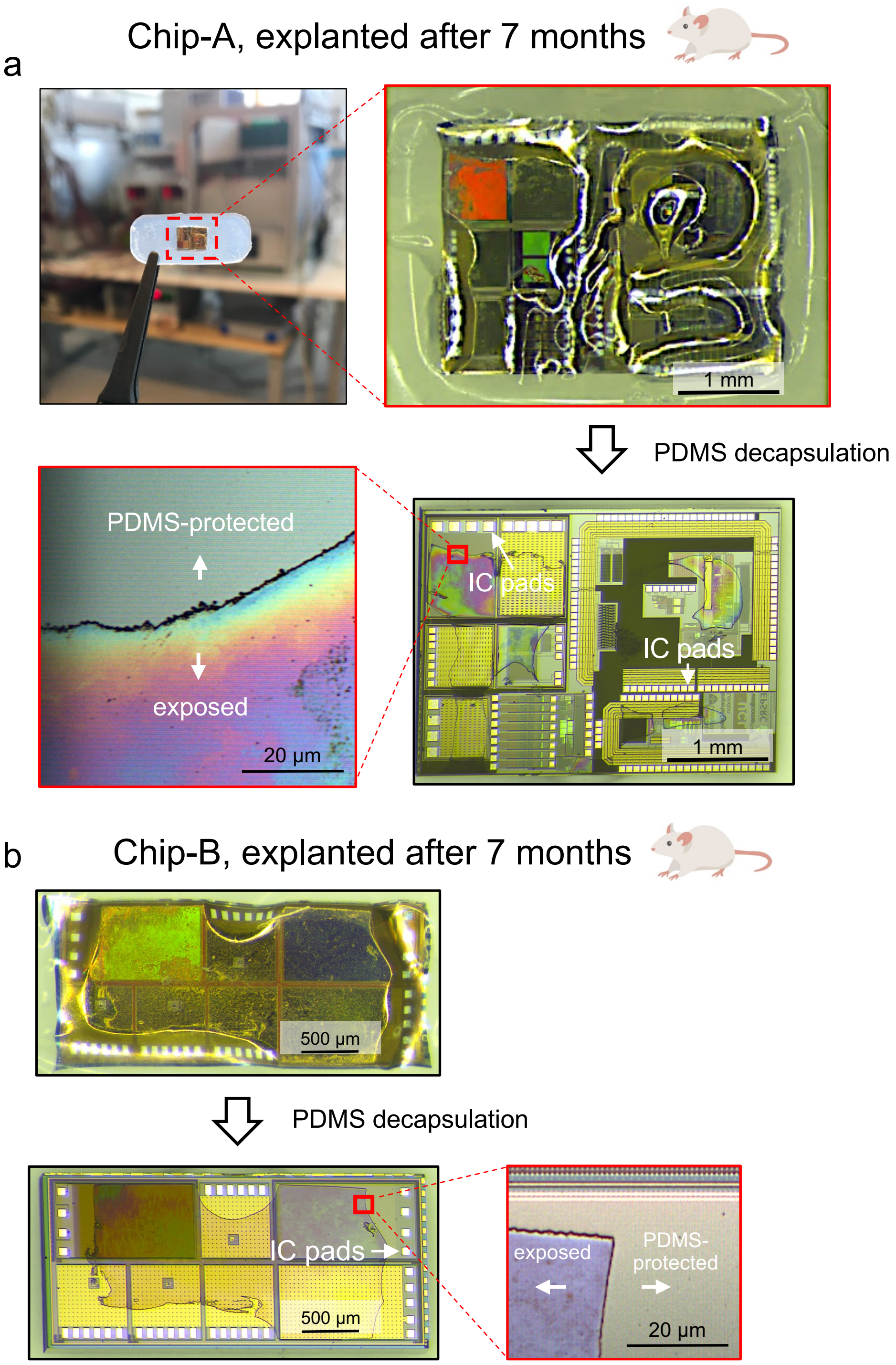

Figure S16. Representative optical micrographs of 7-month explanted chips, before and after PDMS decapsulation. **a)** Optical microscopic image of a Chip-A sample after 7 months of implantation in rat. A noticeable color difference is observed between the PDMS-coated and exposed regions which is due to the *in vivo* degradation of the IC's SiN_X passivation. **b)** Optical micrograph of a Chip-B sample explanted after 7 months. Note that for both Chip-A and B ICs, all the PDMS-coated aluminum pads remained intact.

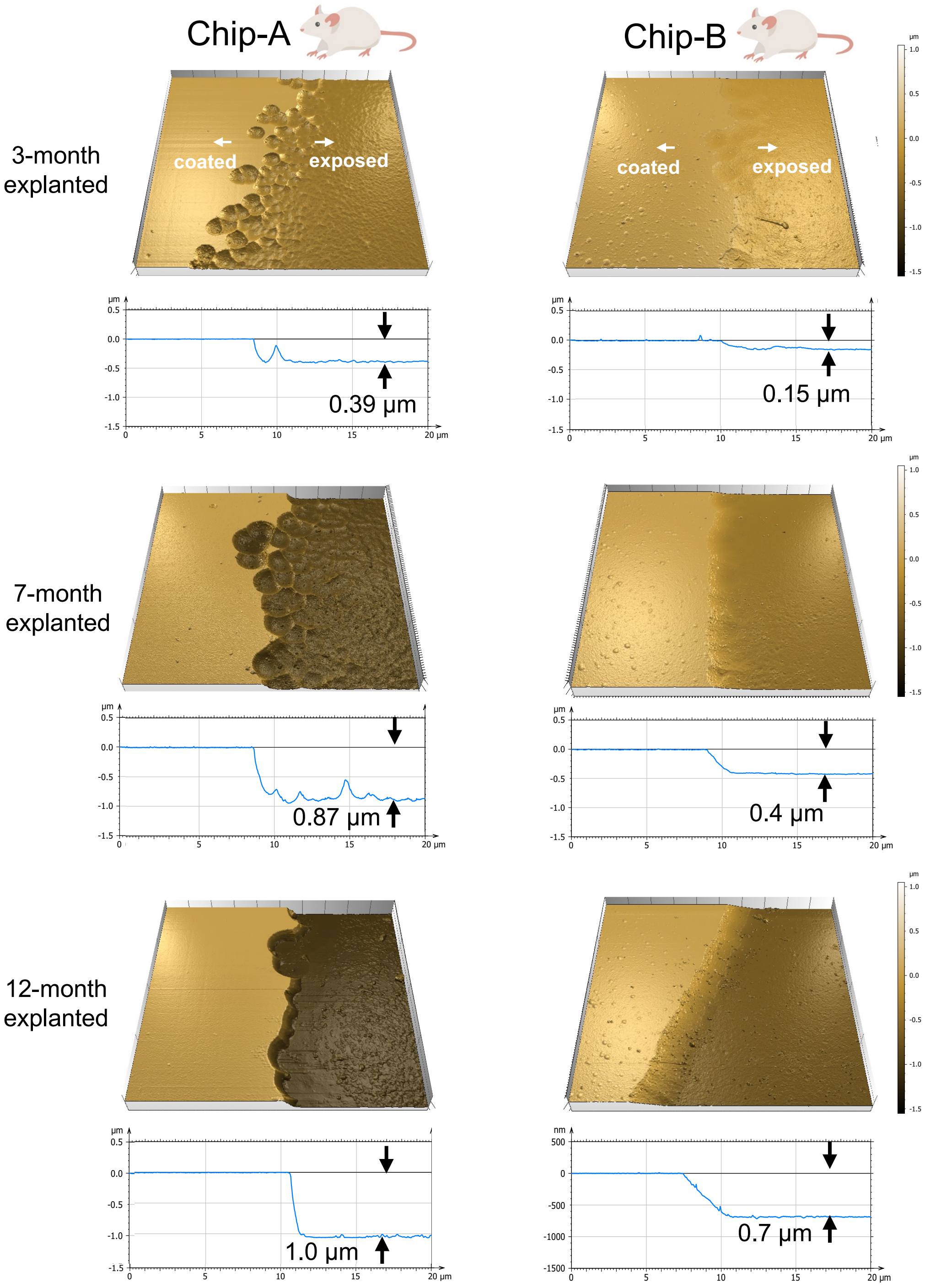

Figure S17. AFM surface topography of explanted ICs analyzed on a 20 µm × 20 µm area at the PDMS-edge after PDMS decapsulation. Chip-A and Chip-B ICs explanted at different time points (3, 7 and 12 month) showing gradual dissolution of the SiN_X layer *in vivo*.

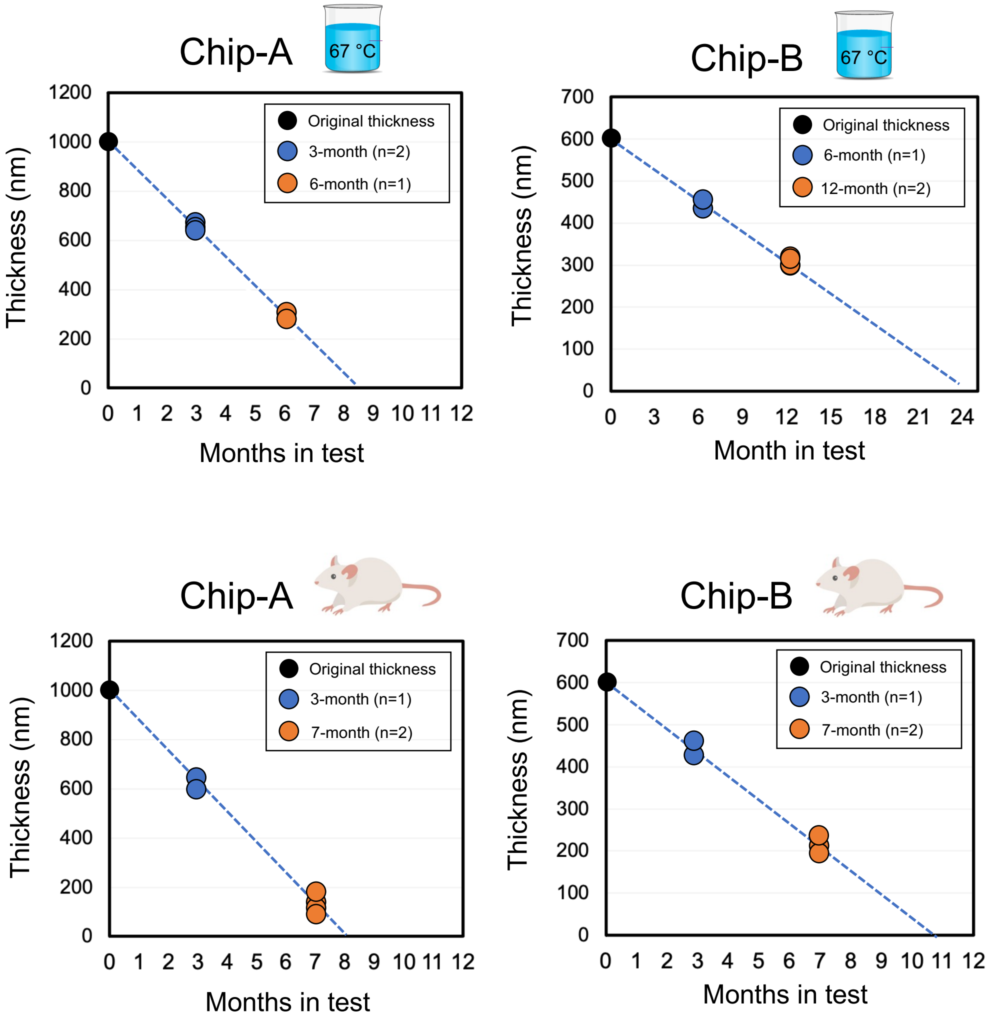

Figure S18. *In vitro* (top)and *in vivo* (bottom) SiN_X passivation loss over time for Chip-A and B ICs. For each sample, two measurements were done at different locations on the chip. Extrapolated line (blue dashed line) shows the expected time points where the entire SiN_X passivation will have dissolved.

Biocompatibility of Silicon-IC passivation layers: SiN_x and SiO_x

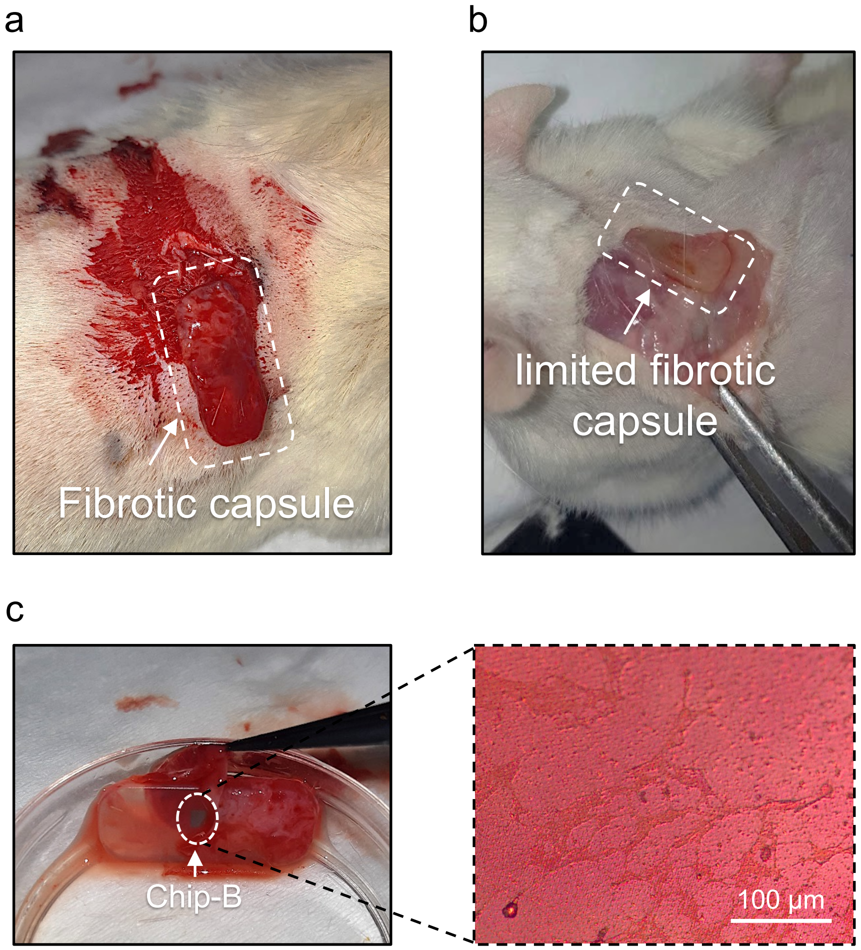

Figure S19. Images of explanted ICs. **a)** Tissue pocket formation around an implanted sample (Chip-A) after three months of implantation. **b)** Explanted sample with limited tissue pocket formation around the sample after 7 months of implantation where the PDMS substrate is clearly visible. **c)** Easy tissue pocket removal from a 7-month explanted Chip-B IC showing no tissue adhesion to the IC passivation surface or the surrounding PDMS. Optical micrograph of the passivation surface showing fibroblasts covering the SiN_x (left).

Biocompatibility of Silicon-IC passivation layers: SiN_X and SiO_X 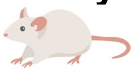

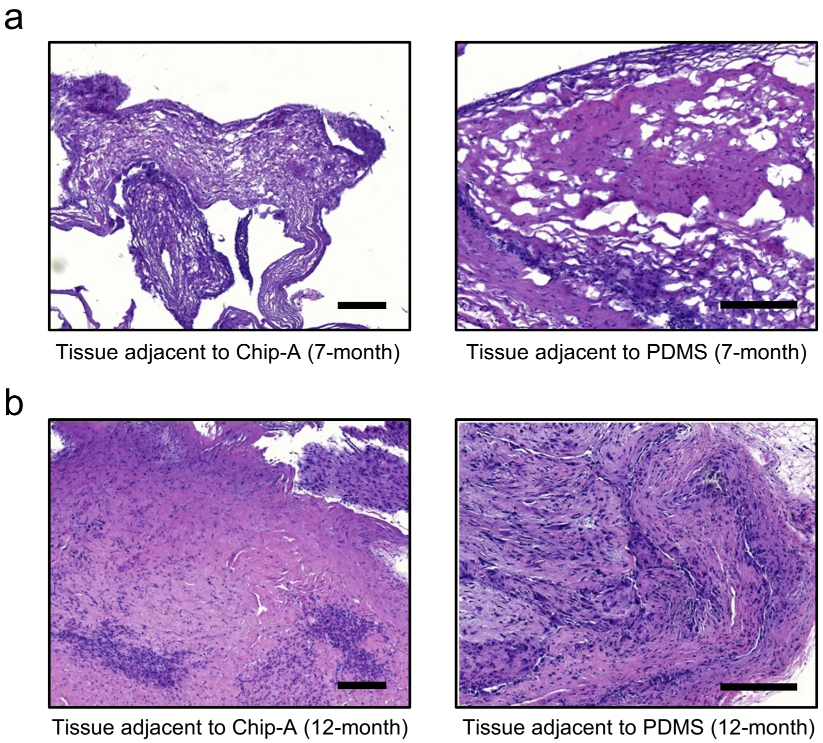

Figure S20. Histology images comparing the hematoxylin and eosin (H&E) stained tissues adjacent to the silicon-IC (Chip-A) and PDMS substate (used as control). **a**) Histology of a 7-month explanted Chip-A. At 7 months, the surface of the IC is SiN_X. **b**) Histology of a 12-month explanted Chip-A. For Chip-A, at month 12, the SiO_X passivation is exposed to tissue for approximately 4 months. Scale bar in all images, 200 µm.

All tissue samples (n=4 for 7-month and n=4 for 12-month) were stained with hematoxylin eosin (H&E) for visualizing the cell nuclei and the cytoplasm. Qualitative analysis of the stained samples showed mature fibrotic tissue with mast cell infiltration in 12-month implants, whereas less mature, more cellular tissue was seen in the case of 7-month implants for both PDMS and silicon-IC implants. Results showed no inflammation or tissue damage after 1-year of subdermal implantation of two CMOS foundry ICs in rats. These results are despite the observed SiN_X dissolution observed for both ICs foundries which suggest the biocompatibility of ICs after long-term implantation.

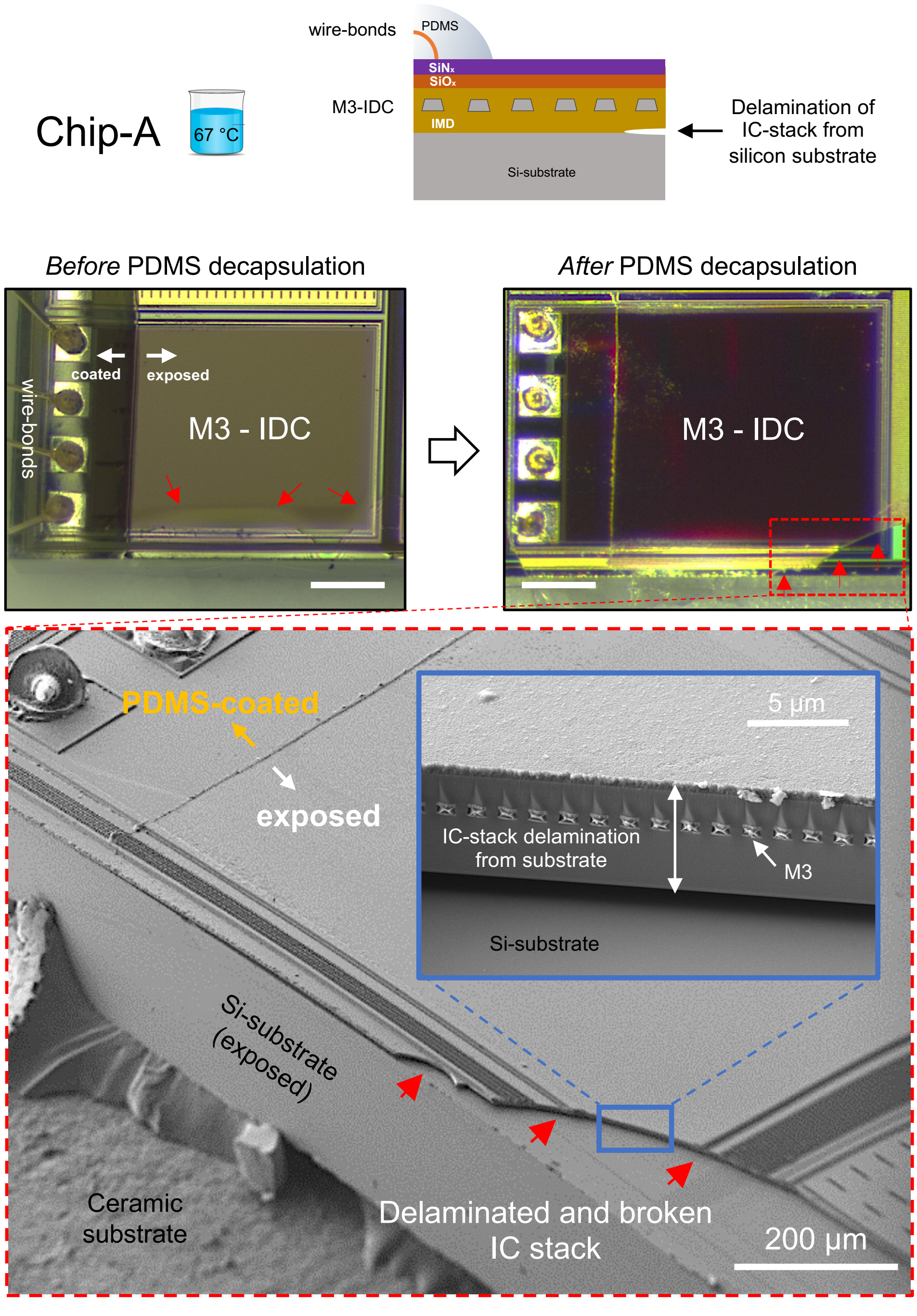

Figure S21. Tilted optical and electron micrographs of a M3-IDC test structure (Chip-A) showing delamination of IC-stack from silicon substrate after 16 months aging in PBS solution at 67 °C . In optical micrographs, delamination is seen as color fringes on the IC edge near the sidewall in the uncoated region, shown with red arrows (left). After PDMS decapsulation, a section of the delaminated area broken off due to handling, shown in red dashed square (right). Scale bar is 200 µm. Bottom image gives a tilted SEM image of the chip sidewall after PDMS-decapsulation. Inset: magnified SEM image from the broken area where delamination of the entire IC stack from the Si-substrate is visible. The metallization used for the M3-IDC test structure is also visible.

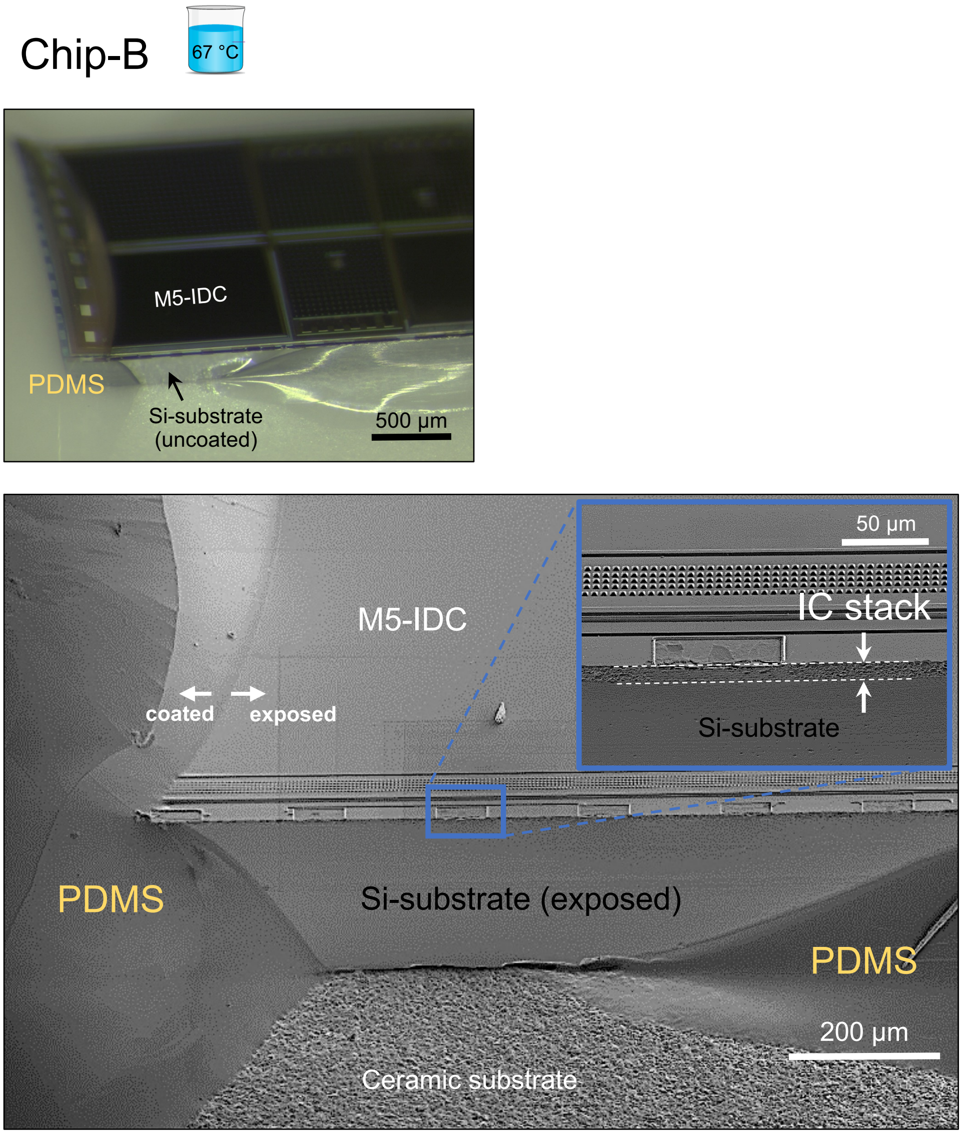

Figure S22. Tilted optical and electron micrograph of a M5-IDC test structure (Chip-B) after 16 months of accelerated aging in 67 °C PBS solution showing no delamination on the chip side wall in the exposed region. Inset: magnified electron image where no delamination is observed between the IC stack (~ 9 μm) and the Si-substrate.

Supplementary Note 6: Accelerated in vitro testing in PBS solution has been extensively investigated as a model to estimate the longevity of novel implantable devices. However, in the exposed bare die regions of the tested ICs, it was found that PBS solution is not an ideal model to mimic the complex in vivo environment as a ~ 20 times higher nitride dissolution rate was observed in the body environment compared to the estimated dissolution rate in PBS solution at 37 °C (assuming an Arrhenius acceleration factor from 37 °C to 67 °C). Using ToF-SIMS analysis, we compared the chemistry of the first few nanometers (0 - 5 nm) of the dissolving SiN_x layers (see figure below). Results showed slight differences in the surface chemistry after exposure to different aging media (PBS at 67 °C and rat). All aged ICs created an off-stochiometric oxidized (SiO_X) layer with high [OH^-] on top of the nitride passivation. ICs exposed to PBS solution created a slightly thicker oxidized layer with higher [SiO_2^-] and lower [OH^-] intensity compared to the ICs exposed to the body. Si-O bonds are thermodynamically more stable than Si-N bonds [6]. Therefore, the created oxidized layer on top of the nitride passivation can act as a protective layer preventing or delaying attack by water molecules. The higher *in vivo* nitride dissolution rate could be due to the thinner and lower quality top oxide layer making the deeper Si-N bonds more readily available for attack and dissolution. The theory of nitride dissolution rate relying on the off-stochiometric oxidation reactions has been proposed before [7] and has been experimentally suggested in this investigation. The thinner oxidized layer on the SiN_X passivation *in vivo* could be due to a similar mechanism observed for the corrosion of titanium metal implants [8], where it was found that the body's proteins and enzymes covering the implant inhibit the surface oxidation of the metal, allowing more bulk metal to be available for corrosion.

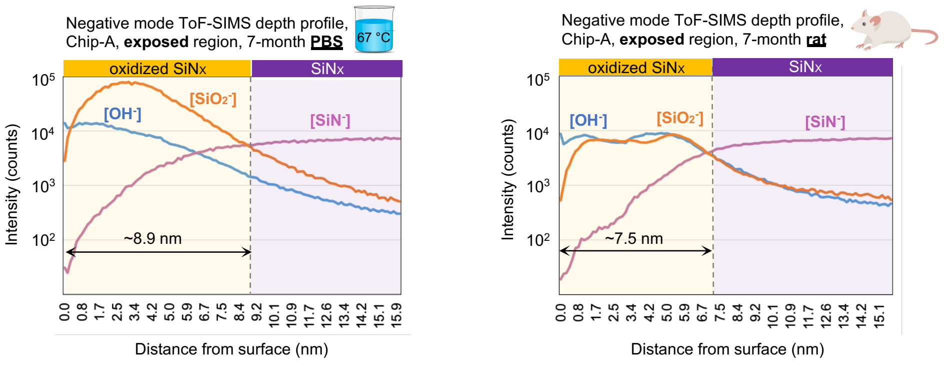

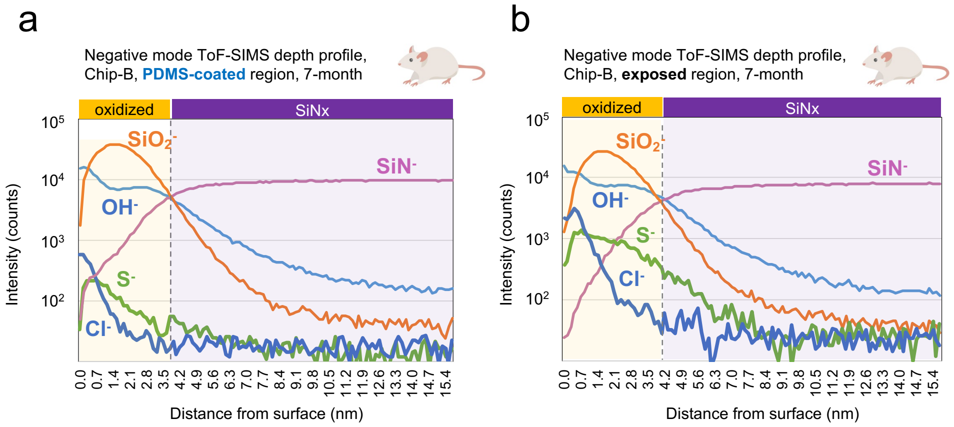

Figure S23. Shallow negative mode ToF-SIMS depth profiles acquired with sub-nanometer step-size (0.135 nm) from 0 - 15 nm from the **(a)** PDMS-coated, and **(b)** exposed (uncoated) regions of a 7-month explanted Chip-B IC.

Supplementary Note 7: Average [SiN^-] intensities within the SiNx passivation bulk were analyzed and are given below (averaged ToF-SIMS depth profile data from 50 - 100 nm).
The SiO_x-bulk was also examined by evaluating the [OH^-] and [SiO_2^-] intensities in the layer (averaging the intensities between 100 - 200 nm). Figure below shows the average intensities for the exposed and PDMS-coated regions after 12 months of exposure to both aging environments. On both Chip-A and B samples, a stable [OH^-] intensity is seen in the SiO_X layer for the PDMS-coated regions. A slight increase in [OH^-] intensity is recorded for the exposed areas for Chip-B. Note that the SiO_X passivation can be directly exposed to the aging environments (either PBS or body) for the exposed (uncoated) regions after the complete dissolution of the top nitride passivation. In the PDMS-coated regions, however, the SiO_X is always protected by both PDMS and the top SiN_X passivation layer. Data in the figure below is presented as average of 4 measurement results, performed on n=2 samples (per chip foundry) with 2 measurements per sample.

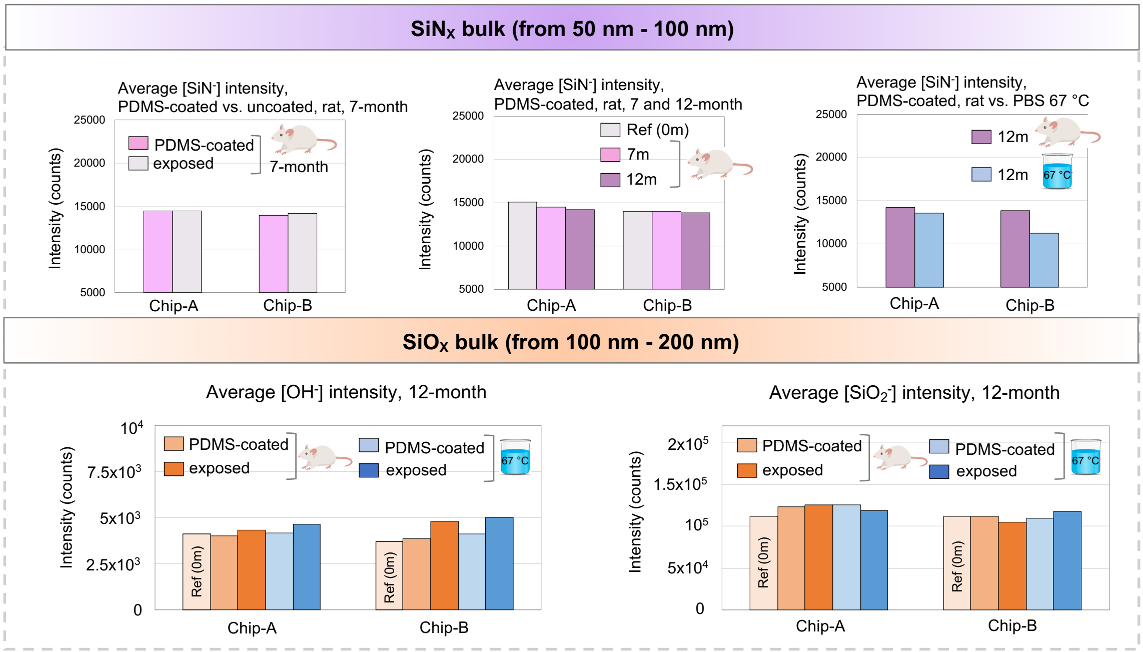

a

Chip-A (15V biased)

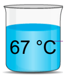

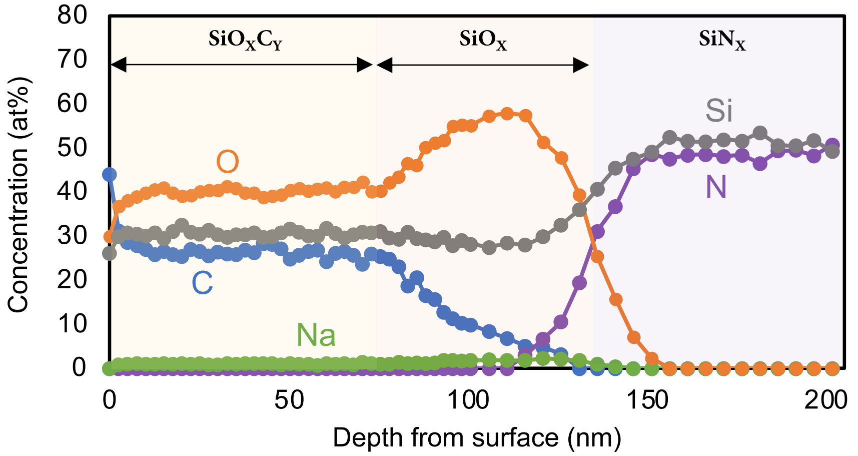

		Si	N	O	C	Na	Cl	K
SiO$_X$C$_Y$	~70 nm	31.4	0	40.5	25.6	0.8	0	0
SiO$_X$	~60 nm	28	0	58	6.8	1.8	0	0.6
SiN$_X$		50	50	0	0	0	0	0

b

Chip-B (15 V biased)

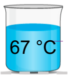

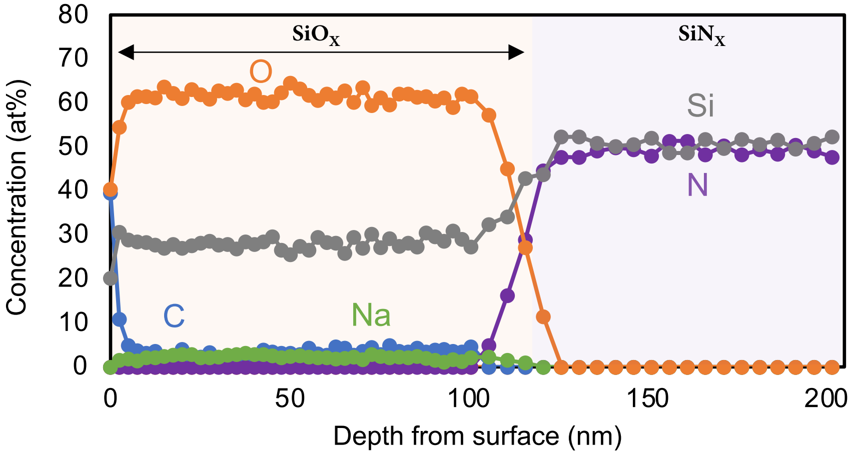

		Si	N	O	C	Na	Cl	K
SiO$_X$	~116 nm	27.9	0	62.1	1.6	2.9	0	0.5
SiN$_X$		50	50	0	0	0	0	0

Figure S24. X-ray photoelectron spectroscopy (XPS) depth profiles giving the chemical composition of the top oxidized layer developed on the SiN$_X$ passivation layers after 12 months of continuous electrical biasing with 15 V DC in PBS solution at 67 °C. **a)** Chemical composition of the developed oxidized layer on Chip-A when biased between M3-IDC (negative) and PBS (positive). **b)** Chemical composition of the developed oxidized layer on Chip-B when biased between M5-IDC (negative) and PBS (positive).

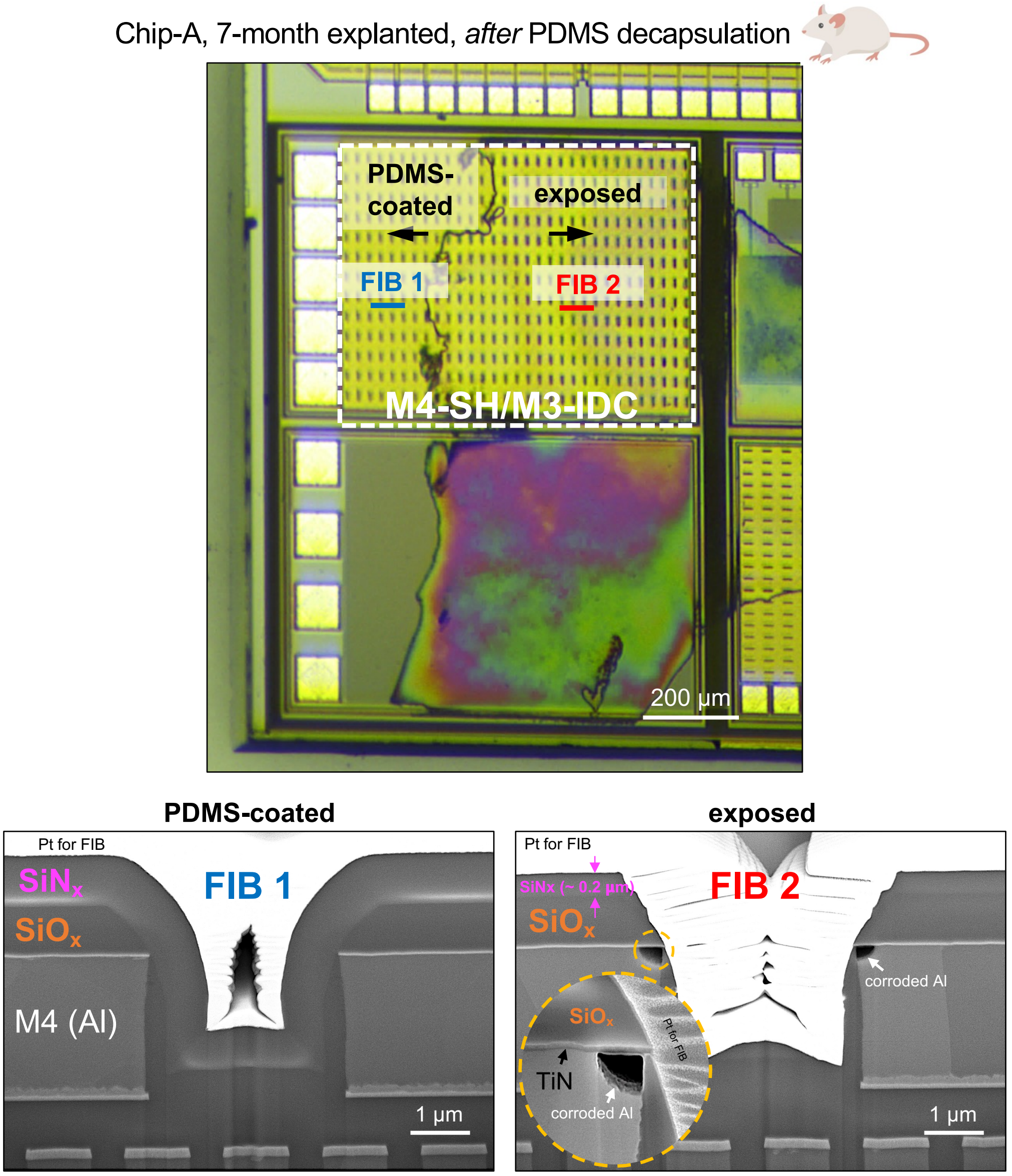

Figure S25. Optical micrograph and SEM cross sectional images of a Chip-A sample explanted at month 7 and PDMS decapsulated. Blue and red lines on the M4-SH/M3-IDC structure indicate FIB cuts in the PDMS-coated and exposed (uncoated) regions, respectively. SEM cross-sectional images of the FIB cuts compare the PDMS-coated (FIB 1: bottom left) and exposed (FIB 2: bottom right) regions of the M4-SH/M3-IDC test structure. In the exposed region, corrosion of the Metal-4 (top metal) aluminum (Al) layer is visible due to the dissolution of the SiN_X passivation and the poor conformality of the SiO_X passivation layer. Note that on top of the IC, in the flat area, a thinned SiN_X is still present. The sides, however, due to poor conformality, have a thinner SiN_X. Inset shows a high magnification of the corroded aluminum (Al) metal while the titanium nitride (TiN) layer remains intact. The poor conformality of the SiN_X and SiO_X passivation layers is a result of using the top metallization (in this case M4).

Supplementary Note 8: As explained in Supplementary Note 1, adhesion between the PDMS and IC surface is crucial for preventing lateral fluid ingress and moisture condensation that could lead to shunt leakage paths. To qualitatively assess adhesion strength and detect any potential debonding after in vitro and in vivo aging, a shear force was applied to the PDMS edge on the 12-month aged IC structures (see figure below). This was performed by using tweezers to apply pressure on the PDMS while microscopically inspecting for any detachment from the IC surface. No separation of PDMS from the IC was observed after aging, even under significant applied pressure. Instead, the shear force primarily led to cohesive breakage within the PDMS.

It should be noted that in the body, PDMS interfaces are subjected to various stress factors, including moisture (which permeates through the PDMS) and mechanical loading. In the accelerated in vitro study, no mechanical loading was applied to the samples. However, PDMS-device interface bonds are also affected by hydrothermal stresses, where the combination of moisture and temperature can accelerate bond degradation. Previous studies have shown that hydrothermal stresses can degrade PDMS-device interfaces at rates that follow the Arrhenius law [9]. Therefore, in the in vitro study, samples soaked at 67 °C experienced chemical stress on their interfaces for approximately eight times longer than they would at body temperature.

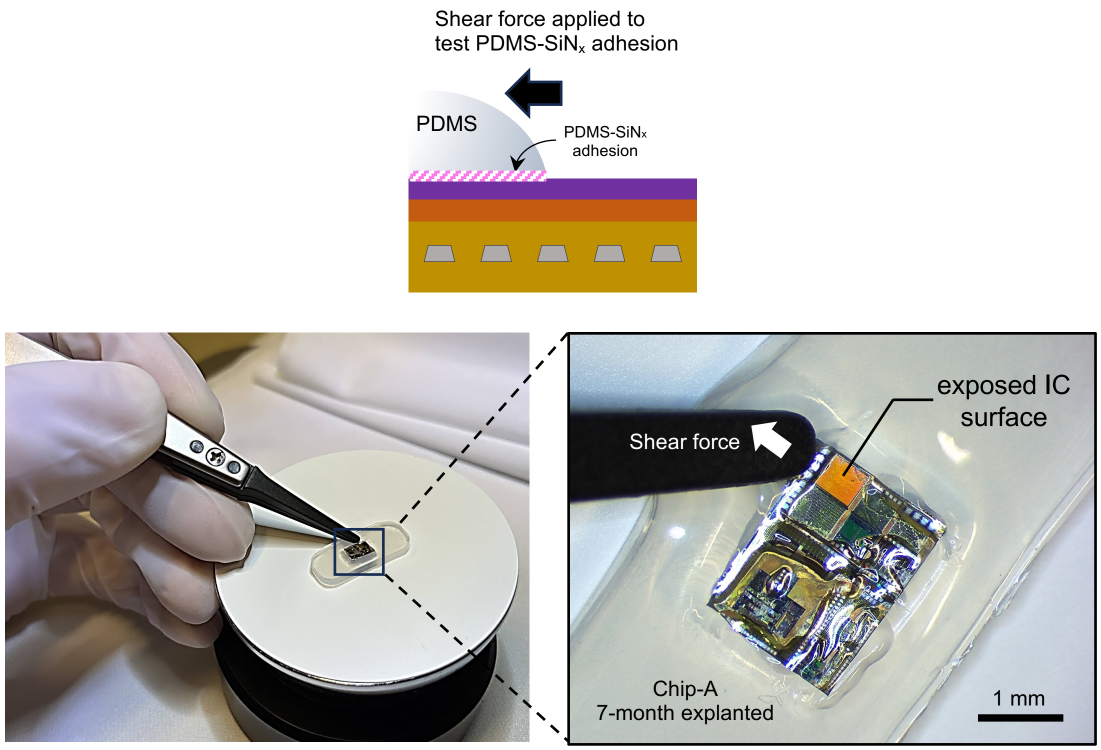

Supplementary References:

[1] Donaldson, N., C. Lamont, A. Shah Idil, M. Mentink, and T. Perkins. "Apparatus to investigate the insulation impedance and accelerated life-testing of neural interfaces." Journal of neural engineering 15, no. 6 (2018): 066034.

[2] Iannuzzi, Melanie. "Bias Humidity Performance and Failure Mechanisms of Nonhermetic Aluminum SIC's in an Enviornment Contaminated with Cl 2." *IEEE Transactions on Components, Hybrids, and Manufacturing Technology* 6, no. 2 (1983): 191-201.

[3] Baylakoğlu, İlknur, Aleksandra Fortier, San Kyeong, Rajan Ambat, Helene Conseil-Gudla, Michael H. Azarian, and Michael G. Pecht. "The detrimental effects of water on electronic devices." *e-Prime-Advances in Electrical Engineering, Electronics and Energy* 1 (2021): 100016.

[4] Birkholz, M., K-E. Ehwald, D. Wolansky, I. Costina, C. Baristiran-Kaynak, M. Fröhlich, H. Beyer, A. Kapp, and F. Lisdat. "Corrosion-resistant metal layers from a CMOS process for bioelectronic applications." Surface and Coatings Technology 204, no. 12-13 (2010): 2055-2059.

[5] Alpern, P. et al. On the way to zero defect of plastic-encapsulated electronic power devicespart III: Chip coating, passivation, and design. IEEE Transactions on Device and Materials Reliability 9, (2009).

[6] Lee, H. I. et al. Degradation by water vapor of hydrogenated amorphous silicon oxynitride films grown at low temperature. Sci Rep 7, (2017).

[7] Pezzotti, Giuseppe, Tetsuya Adachi, Francesco Boschetto, Wenliang Zhu, Matteo Zanocco, Elia Marin, B. Sonny Bal, and Bryan J. McEntire. "Off-Stoichiometric Reactions at the Cell–Substrate Biomolecular Interface of Biomaterials: In Situ and Ex Situ Monitoring of Cell Proliferation, Differentiation, and Bone Tissue Formation." International Journal of Molecular Sciences 20, no. 17 (2019): 4080.

[8] Eliaz, Noam. "Corrosion of metallic biomaterials: a review." Materials 12, no. 3 (2019): 407.

[9] Lonys, Laurent, Anne Vanhoestenberghe, Nicolas Julémont, Stéphane Godet, Marie-Paule Delplancke, Pierre Mathys, and Antoine Nonclercq. "Silicone rubber encapsulation for an endoscopically implantable gastrostimulator." Medical & biological engineering & computing 53 (2015): 319-329.
